# The Zebrafish as a New Model for the *In Vivo* Study of *Shigella flexneri* Interaction with Phagocytes and Bacterial Autophagy

**DOI:** 10.1371/journal.ppat.1003588

**Published:** 2013-09-05

**Authors:** Serge Mostowy, Laurent Boucontet, Maria J. Mazon Moya, Andrea Sirianni, Pierre Boudinot, Michael Hollinshead, Pascale Cossart, Philippe Herbomel, Jean-Pierre Levraud, Emma Colucci-Guyon

**Affiliations:** 1 Section of Microbiology, MRC Centre for Molecular Bacteriology and Infection, Imperial College London, London, United Kingdom; 2 Institut Pasteur, Unité des Interactions Bactéries-Cellules, Département de Biologie Cellulaire et Infection, Paris, France; 3 Inserm, U604, Paris, France; 4 INRA, USC2020, Paris, France; 5 Institut Pasteur, Unité Macrophages et Développement de l'Immunité, Département de Biologie du Développement et des Cellules Souches, Paris, France; 6 CNRS, URA2578, Paris, France; 7 INRA, Virologie et Immunologie Moléculaire, Jouy-en-Josas, France; 8 Section of Virology, Faculty of Medicine, Imperial College London, London, United Kingdom; University of Illinois, United States of America

## Abstract

Autophagy, an ancient and highly conserved intracellular degradation process, is viewed as a critical component of innate immunity because of its ability to deliver cytosolic bacteria to the lysosome. However, the role of bacterial autophagy *in vivo* remains poorly understood. The zebrafish (*Danio rerio*) has emerged as a vertebrate model for the study of infections because it is optically accessible at the larval stages when the innate immune system is already functional. Here, we have characterized the susceptibility of zebrafish larvae to *Shigella flexneri*, a paradigm for bacterial autophagy, and have used this model to study *Shigella*-phagocyte interactions *in vivo*. Depending on the dose, *S. flexneri* injected in zebrafish larvae were either cleared in a few days or resulted in a progressive and ultimately fatal infection. Using high resolution live imaging, we found that *S. flexneri* were rapidly engulfed by macrophages and neutrophils; moreover we discovered a scavenger role for neutrophils in eliminating infected dead macrophages and non-immune cell types that failed to control *Shigella* infection. We observed that intracellular *S. flexneri* could escape to the cytosol, induce septin caging and be targeted to autophagy *in vivo*. Depletion of p62 (sequestosome 1 or SQSTM1), an adaptor protein critical for bacterial autophagy *in vitro*, significantly increased bacterial burden and host susceptibility to infection. These results show the zebrafish larva as a new model for the study of *S. flexneri* interaction with phagocytes, and the manipulation of autophagy for anti-bacterial therapy *in vivo*.

## Author Summary

Autophagy, an ancient and highly conserved intracellular degradation process, is viewed as a critical component of innate immunity because of its ability to deliver cytosolic bacteria to the lysosome. However, a complete understanding of the molecules and mechanisms restricting cytosolic bacteria has not been obtained, and the role of bacterial autophagy *in vivo* remains poorly understood. *Shigella flexneri* are human-adapted *Escherichia coli* that have gained the ability to invade the colonic mucosa, causing inflammation and diarrhea. The intracellular lifestyle of this pathogen has been well-studied *in vitro*, and *Shigella* has recently gained recognition as a paradigm of bacterial autophagy. We show that the zebrafish larva represents a valuable new host for the analysis of *S. flexneri* infection. Interactions between bacteria and host phagocytes can be imaged at high resolution *in vivo*, and the zebrafish model should prove useful for understanding the cell biology of *Shigella* infection. We use zebrafish larvae to investigate the role of bacterial autophagy in host defense, and observed that the perturbation of autophagy can adversely affect host survival in response to *Shigella* infection. Therefore, the zebrafish constitutes a valuable system to develop new strategies aimed at pathogen clearance by manipulation of anti-bacterial autophagy.

## Introduction

Macroautophagy (hereafter referred to as autophagy) is an intracellular degradation process by which cytosolic materials are delivered to the lysosome. The canonical autophagy pathway involves the initiation and elongation of double-membrane autophagosomes to sequester cargo, and this process requires 36 autophagy-related (ATG) proteins conserved from yeast to man [1]. Autophagy has diverse functions in important cellular processes such as development, aging and inflammation, and is also linked to a wide range of disease states including microbial infection [2], [3]. By binding to ATG8 family proteins and delivering them to recognized cargo, autophagy receptors can mediate selective targeting of intracellular bacteria to autophagy [4], [5]. p62 (sequestosome 1 or SQSTM1) is a well-characterized autophagy receptor [6] and belongs to a newfound category of pattern recognition receptors called SLRs (sequestosome 1/p62-like receptors) linking autophagy to innate immunity [7]. Discovered almost 10 years ago, bacterial autophagy has been highlighted as a fundamental host cell response to bacterial invasion *in vitro* by degrading intracellular pathogens including *Shigella flexneri* [8], *Listeria monocytogenes* [9], *Salmonella* Typhimurium [10] and *Mycobacterium tuberculosis* [11]. Since then, research in the field has exploded, revealing that some pathogens may avoid autophagy-mediated degradation while others may exploit the autophagy machinery for intracellular survival [12], [13]. However, few *in vivo* studies have been performed and, as a result, the consequence of bacterial autophagy on disease outcome remains obscure.

*S. flexneri* are human-adapted *Escherichia coli* that have gained the ability to invade the colonic mucosa, causing inflammation and diarrhea. The intracellular lifestyle of this pathogen has been well-studied *in vitro*, and *Shigella* has recently gained recognition as a paradigm of bacterial autophagy [8], [12]–[16]. Once in the cytosol, the actin-based motility of *Shigella* is counteracted by septin cage-like structures that target bacteria to p62-mediated autophagy [14], [15]. Septins are GTP-binding proteins that form higher-order structures including filaments and rings, and are viewed as a distinct component of the cytoskeleton [17]. The precise role of septins in autophagy is unknown, yet work has shown that septins may help to scaffold the autophagy machinery around actin-polymerizing bacteria [14], [15]. Now, a major issue is to demonstrate the significance of these molecular and cellular events *in vivo* using relevant animal models.

To explore the innate immune response to *Shigella*, several infection models have been useful [18], [19], however, these mammalian models remain poorly suited to image the cell biology of *Shigella* infection *in vivo*. The zebrafish has recently emerged as a non-mammalian vertebrate model to study the development and function of the immune system [20], [21]. It is a genetically tractable organism, sharing many immune pathways and cell types with mammals [22]. The natural translucency of zebrafish larvae enables non-invasive *in vivo* imaging of individual cells and microbe-phagocyte interactions at high resolution throughout the organism [23]–[32]. While zebrafish larvae have been used to study infection by many different bacteria ([23]–[32], reviewed in [33]), including *E. coli* [26], *L. monocytogenes* [24] and *M. marinum* [25], *Shigella* infection has not yet been studied in this model. Considering that, at the cellular level, the infectious process for *S. flexneri* is similar to that of *L. monocytogenes* and *M. marinum*, i.e., these bacteria escape from the phagocytic vacuole to the cytosol where they form actin tails or are recognized by autophagy [15], [34]–[36], we chose to investigate the outcome of experimental shigellosis and the role of autophagy in zebrafish larvae.

We first established that *Shigella* is pathogenic for zebrafish larvae, and characterized the lethal dose and kinetics of the infection following microinjection. Inoculated *S. flexneri* were rapidly engulfed by macrophages and neutrophils, and these events could be captured in real time, highlighting a scavenger role for neutrophils in eliminating infected macrophages and non-immune cell types that have failed to control *Shigella* infection. Strikingly, the number of both macrophages and neutrophils dramatically decreased in larvae unable to control *Shigella* proliferation, and leukocyte depletion was associated with bacteremia preceding the death of the larvae. We also observed that intracellular *S. flexneri* could escape to the cytosol, induce septin caging and be targeted to autophagy *in vivo*. We then used *Shigella* infection of the zebrafish to study the role of bacterial autophagy *in vivo*, and showed that the depletion of p62-mediated autophagy significantly increased bacterial burden and zebrafish mortality. These data highlight the zebrafish model to study *S. flexneri* interaction with phagocytes *in vivo*. Moreover, the zebrafish constitutes a valuable system to develop new strategies aimed at pathogen clearance by manipulation of anti-bacterial autophagy.

## Results

### *Shigella flexneri* can invade zebrafish cells, escape from the phagosome and induce septin caging at 28°C

**Figure 1:**
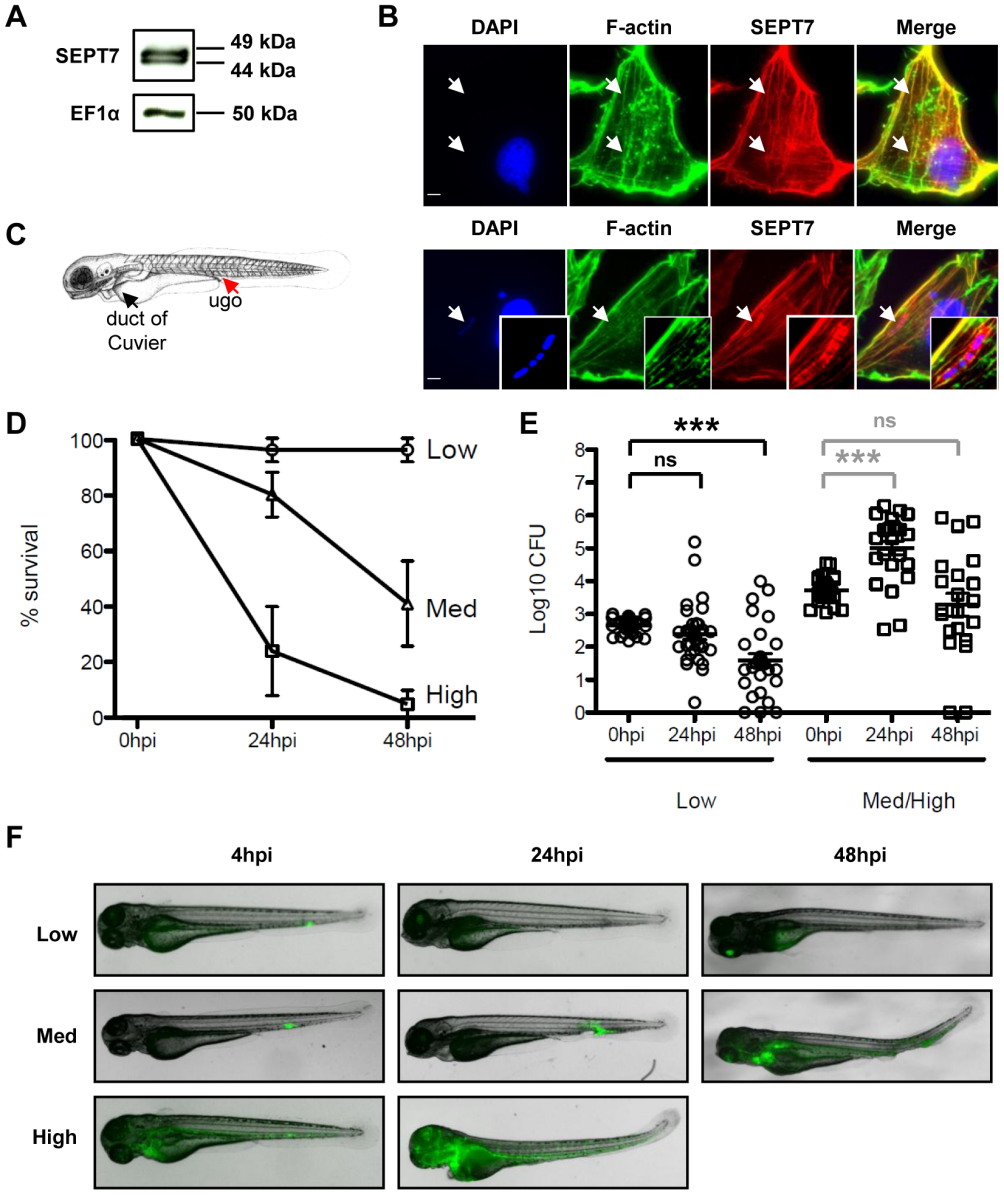
Characterization of zebrafish cells and infection with *Shigella*. A. Western blots of larval extracts, using antibodies against SEPT7 or elongation factor 1-α (EF1α). See Fig. S1B for complete lane of SEPT7 blot and antibody specificity. B. Immunofluoresence microscopy of ZF-AB cells. F-actin (green), SEPT7 (red), DAPI (blue). Top: uninfected cell; note colocalization of actin and SEPT7 filaments (arrows). Bottom: *Shigella*-infected cell; note septin cage-like structure surrounding DAPI-labeled *Shigella* (arrow). Scale bar, 4 µm. C. Scheme of a 72 hpf larva (length ∼3.5 mm), showing the duct of Cuvier (the wide vessel flowing over the yolk; black arrowhead), also called common cardinal vein, and the iv injection site (red arrowhead) in the tail vein just caudal to the urogenital opening (ugo). D. Survival curves of 72 hpf larvae injected with various doses of *S. flexneri* and incubated at 28°C for 48 hpi. The effective inoculum, quantified a posteriori, was classified as low (<10^3^ CFU, open circles; effective range: 1.5–9.9×10^2^ CFU), medium (∼4×10^3^ CFU, open triangles; effective range: 1.3–6.0×10^3^ CFU) or high (∼10^4^ CFU, open squares; effective range: 7.8–34.2×10^3^ CFU). Mean±SEM of n = 10–24 larvae per group from 3 or more independent experiments per inoculum class. E. Enumeration of live bacteria in homogenates from individual larvae at various times post infection measured by plating onto LB. Low dose inoculum = open circles. Medium or high dose inoculum = open squares. Of note, dead larvae at 24 or 48 hpi (overwhelmed with bacteria, as determined by fluorescence stereomicroscopy) were not included here, and only larvae having survived the infection (thus far) were included here (and in this case, statistics are in grey). Mean±SEM also shown (horizontal bars). Significance testing performed by Student's t test. ns, P>0.05; ***, P<0.001. F. Distribution of bacteria (GFP-*Shigella*) determined by live imaging using a fluorescence stereomicroscope at various times post injection of a low, medium or high dose inoculum. Overlay of transmission image (grey) and GFP fluorescence (green).

The infection cycle of *Shigella* in human cells is well understood. After internalization, *Shigella* escape from the phagocytic vacuole to the cytosol where they may be recognized by septin cages and autophagy [14], [15]. As *Shigella* is not a natural pathogen of fish and grows optimally at 37°C, initial experiments sought to determine whether the hallmarks of the *Shigella* infection cycle - invasion, phagosome escape and cytoskeleton reorganization- could be reproduced at 28°C, the optimal growth temperature of zebrafish. To test this, we first established a zebrafish fibroblast-like cell line (ZF-AB; see Material and Methods) that we could infect *in vitro*. The septins are a family of proteins required for the autophagy of *Shigella* in human cells, and SEPT7 in particular is essential for mammalian septin function [17]. Pan et al. reported a *sept7* orthologue in zebrafish [37], and our own search of the most recent zebrafish genome assembly (zv9, www.ensembl.org) and EST databases identified three genes encoding proteins highly similar to the human protein (Fig. S1A), with predicted molecular weights of 48–49 kDa. This close similarity indicated that an antibody raised against human SEPT7 was likely to cross-react with the zebrafish proteins. Indeed, using a human SEPT7 antibody, Western blotting of zebrafish lysates revealed proteins at 49 and 44 kDa (Fig. 1A and S1B), and immunofluorescent labeling of ZF-AB cells revealed septin filaments partially overlapping with the actin cytoskeleton (Fig. 1B), in agreement with what is known in mammalian cells [38], [39]. We then infected ZF-AB cells with *S. flexneri* M90T. As observed with the human epithelial cell line HeLa infected at 28°C (Fig. S1C) and at 37°C [14], [15], *Shigella* invaded ZF-AB cells and induced the formation of septin cages (Fig. 1B), demonstrating that *Shigella* can enter the cytosol of zebrafish cells and be targeted to autophagy. These results strongly suggest that the virulence factors required for *Shigella* invasion, escape from the phagocytic vacuole and replication in the cytosol can be expressed and are functional at 28°C, the most commonly used temperature for zebrafish rearing.

### Zebrafish larvae are susceptible to intravenous *S. flexneri* infection

We next sought to establish whether *S. flexneri* could cause disease in zebrafish swimming larvae. Larvae aged 72 h post fertilization (hpf) were microinjected intravenously (iv) near the cloaca (ugo) with a range of doses of wild-type *S. flexneri* (Fig. 1C), and their survival at 28°C was assessed by regular observation with a stereomicroscope. For *Shigella* doses 10^3^ CFU or less, there was 100% survival of the infected larvae; in contrast, a typical inoculum of 4×10^3^ *Shigella* resulted in the death of most larvae within 48 hours post infection (hpi) (Fig. 1D). Of note, larvae injected with a comparable (or even higher) inoculum of type III secretion system (T3SS) deficient (T3SS−) *Shigella* (Δ*mxiD* strain; Fig. S1D) or the closely related, but non-pathogenic, *E. coli* K12 (data not shown and [26]) always survived for the entire time of observation.

We followed the progression of infection using two approaches. First, to monitor the replication of bacteria in the host, we measured the amount of viable bacteria within larvae at various time points by plating serial dilutions of homogenates of euthanized animals onto bacterial culture dishes. As shown in Fig. 1E, the survival of the larvae was closely associated with the clearance of the inoculated bacteria. Larvae inoculated with a low dose (<10^3^ CFU) progressively cleared bacteria, with a burden reduced by >90% within 48 hrs. In contrast, in larvae inoculated with a high dose, *S. flexneri* numbers increased ∼30fold in the first 24 h; by 48 hpi, most larvae were dead, although in a minority of survivors the bacterial load declined. During infections using comparable or higher inocula of T3SS− *Shigella* (Fig. S1E) or *E. coli* (data not shown and [26]), bacterial numbers always decreased over time, with only a few viable bacteria able to persist for 48 h at highest doses.

**Figure 2:**
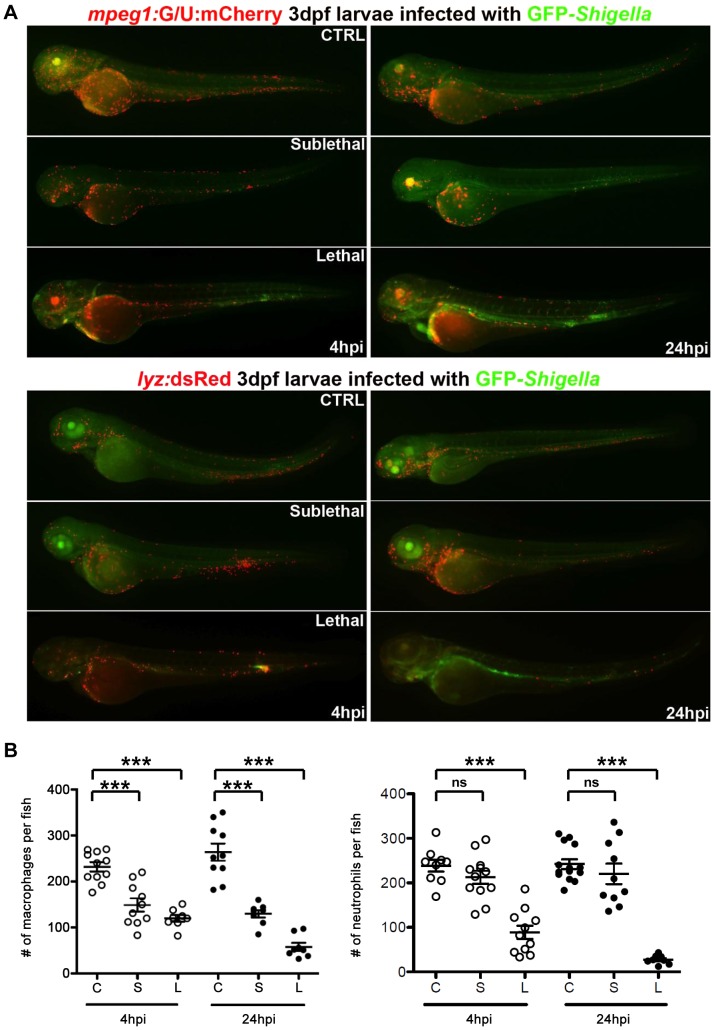
Macrophage and neutrophil depletion upon *Shigella* infection. A. *mpeg1*:G/U:mCherry (red macrophages) and *lyz*:dsRed (red neutrophils) 3 dpf larvae were infected with sublethal or lethal GFP-*Shigella* inocula, fixed 4 and 24 hpi and leukocyte and bacteria labeled using anti-dsRed (red) and anti-GFP (green) antibodies. 4 and 24 hpi uninfected (CTRL), sublethal and lethal inoculated *mpeg1*:G/U:mCherry and *lyz*:dsRed fish are shown. Fixed, labeled whole larvae were imaged using a fluorescent stereomicroscope. Overlay of green and red fluorescence. B. Macrophage (left) and neutrophil (right) counts in uninfected (control, C) or upon sublethal (S) or lethal (L) *Shigella* injections. Macrophages and neutrophils were counted from images using ImageJ and plotted as specified in Material and Methods. Mean±SEM also shown (horizontal bars). Significance testing performed by ANOVA with Bonferroni posttest. ns, P>0.05; ***, P<0.001.

**Figure 3:**
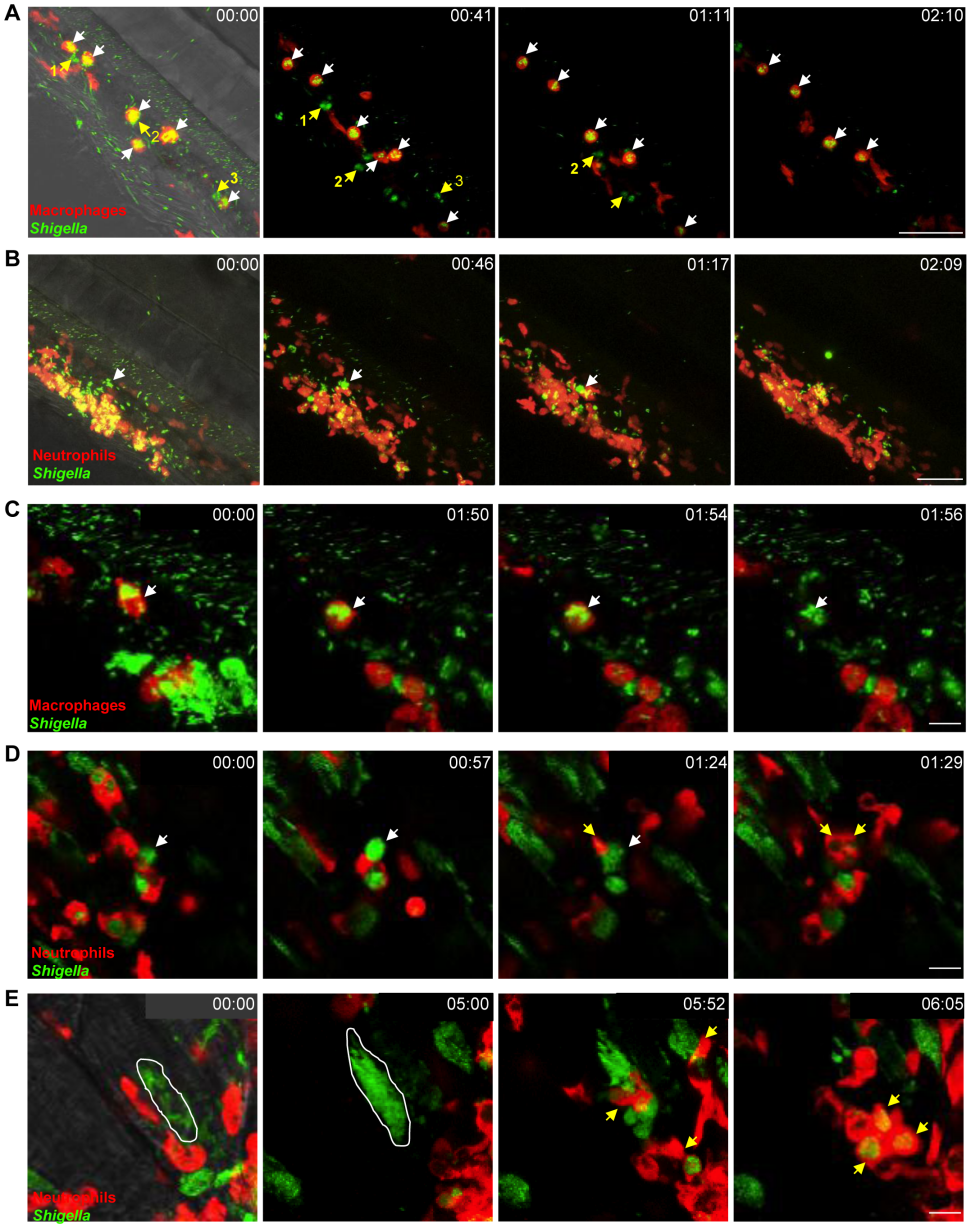
*In vivo Shigella*-phagocyte interactions. A–E: Frames extracted from *in vivo* time-lapse confocal imaging sessions of 3 dpf larvae injected in the bloodstream and in the adjacent mesenchyme with sublethal inocula of GFP-*Shigella*. Caudal area, rostral to bottom right, dorsal to top right. Overlay of green (*Shigella*) and red (macrophages or neutrophils) fluorescence; transmission image (grey) is also overlaid on the first frame in A, B and E as an anatomical guide. Scale bars: A–B, 50 µm; C–E, 10 µm. A. *mpeg1*:G/U:mCherry larva (red macrophages); first frame at 20 mpi. By 20 mpi, *Shigella* have already adhered to or have been engulfed by macrophages (red cells, white arrows). They persist in macrophages over time (last frame at 2h30pi). Note that neutrophils (highlighted in green by the engulfed GFP-*Shigella*, #1,2,3 yellow arrows), that have engulfed bacteria in the mesenchyme have a similar bacterial load to that of macrophages, and efficiently kill the engulfed bacteria (as indicated by the diffuse GFP staining over time in these neutrophils). Maximum intensity projection from 25 planes every 2 µm. See also Video S1. B. *lyz*:dsRed larva (red neutrophils), first frame at 20 mpi. Neutrophils are rapidly recruited to engulf and degrade GFP-*Shigella* in the mesenchyme. By contrast, note the macrophage that remains strongly decorated by GFP-*Shigella* over time (white arrow), highlighting bacterial persistence inside macrophages. Maximum intensity projection from 25 planes every 2 µm. See also Video S4. C. *mpeg1*:G/U:mCherry larva, first frame at 20 mpi. A red macrophage harboring GFP-*Shigella* over time (white arrow) eventually undergoes cell death (loss of red label). Note in the bottom right corner of the field a group of neutrophils that have engulfed the bacteria in the mesenchyme and appear engorged by them, is able to control the engulfed GFP-*Shigella* over time (diffuse and decreasing in intensity GFP signal). Maximum intensity projection of 6 planes every 2 µm. See also Video S5. D. *lyz*:dsRed larva, first frame at 4h30pi. Neutrophils collect dying infected macrophages. GFP-*Shigella* proliferate inside a macrophage (white arrow), which then undergoes cell death, and is quickly engulfed by dsRed+ neutrophils (yellow arrows). Single confocal plane. See also Video S6. E. *lyz*:dsRed larva, first frame at 7 hpi. *Shigella* are able to invade other cell types and replicate in the cytoplasm, causing their death, and subsequent engulfment by neutrophils. The white line delineates a muscle fiber invaded by GFP-*Shigella*. Shortly before 6 h of time-lapse, the muscle cell bursts and is engulfed by red neutrophils (yellow arrows). Maximum intensity projection of 4 planes every 2 µm. See also Video S7.

**Figure 4:**
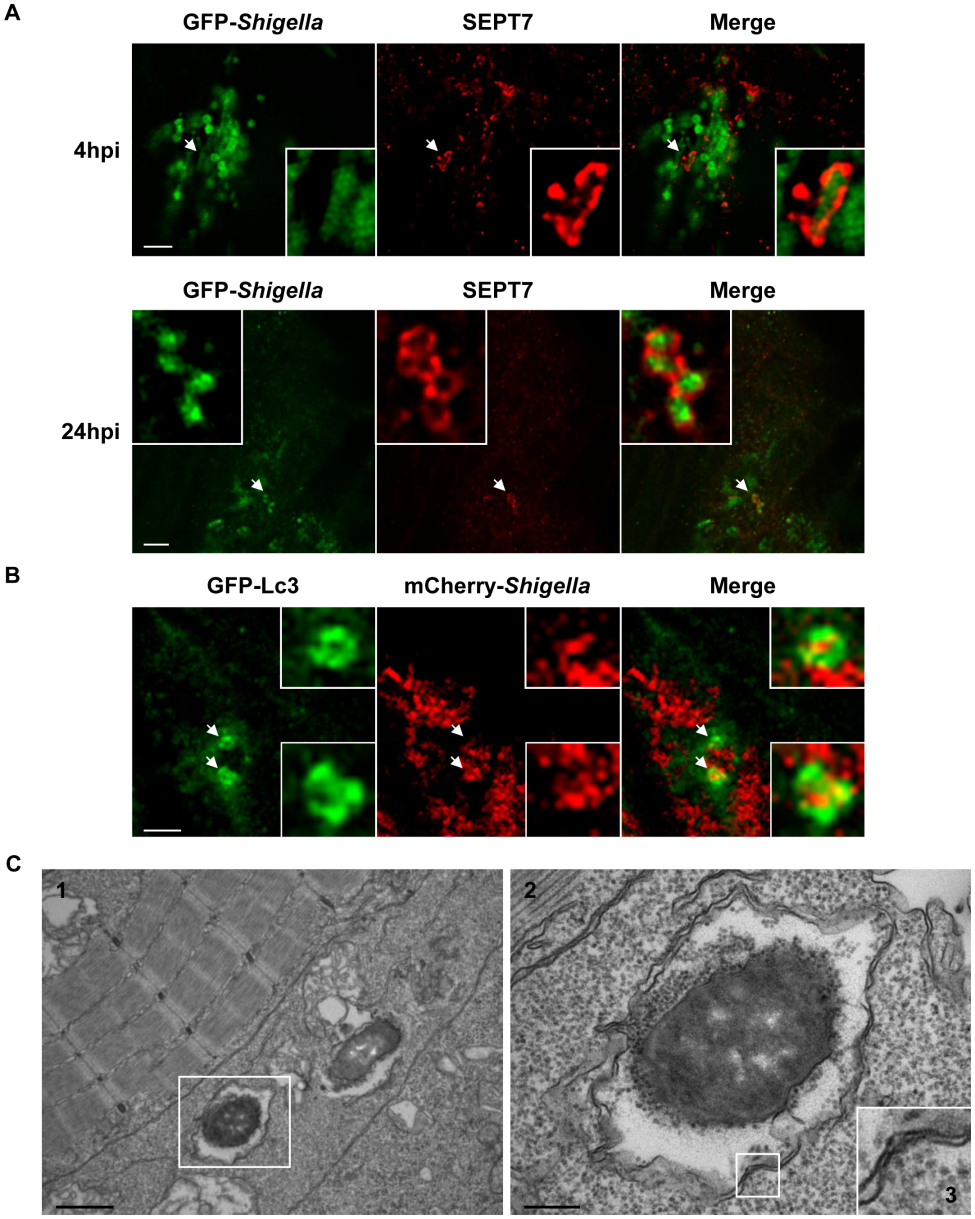
*Shigella* escape to the cytosol and induce septin caging and autophagy in zebrafish larvae *in vivo*. A. Zebrafish larvae were infected in the tail muscle with GFP-*Shigella* for 4 (medium dose) or 24 h (low dose), fixed, labeled with antibodies against SEPT7 (red) and to GFP (green) and imaged by confocal microscopy. Scale bar, 5 µm. B. GFP-Lc3 zebrafish larvae were infected with mCherry-*Shigella* for 4 (medium dose), fixed, labeled with antibodies against mCherry (red) and to GFP (green) and imaged by confocal microscopy. Scale bar, 5 µm. Shown here is an example of a GFP-Lc3 positive cell controlling bacterial replication (better than its neighbouring cells) 4 hpi. See also Video S8 for live imaging observations of GFP-Lc3 recruitment to *Shigella*. C. Cytosolic *Shigella* are sequestered in autophagosomes *in vivo*. Zebrafish larvae were infected in the tail muscle with GFP-*Shigella* for 4 h (medium dose) and fixed for electron microscopy. C2 is an expanded region of boxed region in C1 (i.e., autophagosome sequestering bacteria), C3 is an expanded region of boxed region in C2 (i.e., double membrane, a hallmark of autophagosomes). Scale bar, 1 µm (C1) or 0.25 µm (C2). See also Video S9 for a tilt series of *Shigella* autophagosomes *in vivo*.

As a second approach to follow the progression of infection, larvae were infected with sublethal or lethal inocula of fluorescent *S. flexneri* M90T, i.e. GFP-*Shigella*, and were analyzed by fluorescence microscopy. Large clusters (∼10^2^ bacteria or more) were detectable using a fluorescence stereomicroscope (Fig. 1F, see also Fig. 2 below), while smaller groups and single bacteria could be viewed at higher magnifications using widefield or confocal fluorescence microscopy (see below e.g., Fig. 3, 4). At 4 hpi with a sublethal inoculum of *S. flexneri*, bacterial aggregates, probably associated to phagocytes [26], [40], were observed mostly in the caudal hematopoietic tissue (CHT) near the injection site, as well as in the more distant common cardinal vein over the yolk sac; the majority of the bacteria were cleared from the blood (Fig. 1F, see also Fig. S2 below). The situation was relatively similar in larvae inoculated with a lethal inoculum at 4 hpi, except for the higher number of bacterial foci observed in the CHT and over the yolk sac (Fig. 1F see also Fig. S2 below). However, the infection course by 24 hpi was radically different between the sublethal and lethal inocula. At this time point, larvae that received a sublethal inoculum had almost all cleared the infection, showing few foci of *Shigella* mainly located near the injection point in the caudal part of the larvae. In contrast, larvae that received a lethal inoculum showed a massive infection at 24 hpi, with *Shigella* abundant in the blood (i.e., bacteremia) and in the tissues near the site of injection (Fig. 1F, see also Fig. S2 below).

We observed that larvae that controlled the bacterial proliferation within 24 hpi usually survived. By comparison, inoculation of fluorescent T3SS− *Shigella* or *E. coli* yielded an initial distribution of bacteria (at 4 hpi) very similar to that of wildtype *Shigella*. However, neither T3SS− *Shigella* (data not shown) nor *E. coli* (data not shown and [26]) were proliferating and bacterial numbers always decreased over time (in agreement with CFU enumeration, Fig. S1E). These data strongly suggest that virulence factors required for *Shigella* pathogenesis are expressed during infection of zebrafish larvae at 28°C. Actin tail and septin cage formation are critically dependent upon expression of *Shigella* virulence factors *icsA* (T3SS independent) and *icsB* (T3SS dependent) [14], [15]. By extracting RNA from zebrafish larvae infected with *Shigella*, the expression of *icsA* and *icsB* were clearly detected at 4, 12 and 24 hpi (Fig. S1F). In agreement with observations from survival assays (Fig. 1D), CFU enumerations (Fig. 1E) and live confocal fluorescence microscopy (see below, e.g., Video S1, S3, S5), these data indicate that *Shigella* virulence factors are expressed at 28°C during infection of zebrafish larvae. They also show that zebrafish larvae are susceptible to *S. flexneri*, suggesting their applicability to study the innate immune response to *Shigella* infection.

### Macrophage and neutrophil behaviour upon *Shigella* infection

To analyse macrophage and neutrophil behaviour *in vivo* upon *Shigella* infection, transgenic zebrafish larvae (3 dpf) harboring red macrophages (Tg(*mpeg1*:Gal4-FF)*^gl25^*/Tg(UAS-E1b:nfsB.mCherry)*^c264^*, herein referred as *mpeg1*:G/U:mCherry) or red neutrophils (Tg(*lyz*:dsRed)*^nz50^*, herein referred to as *lyz*:dsRed) were inoculated with sublethal or lethal inocula of GFP-*Shigella*, and macrophages and neutrophils upon *Shigella* infection were followed *in vivo* using widefield fluorescence microscopy. Strikingly, the number of both macrophages and neutrophils appeared to decrease strongly in larvae unable to control *Shigella* proliferation, and leukocyte depletion was associated with bacteremia preceding larval death (Fig. 2A, S2). To more precisely quantify macrophages and neutrophils, we fixed larvae at 4 or 24 hpi, stained them with anti-dsRed and anti-GFP antibodies to label leukocytes and GFP-*Shigella* respectively, and counted the number of labeled macrophages and neutrophils per larva (Fig. 2B). Macrophage counts significantly decreased both in lethally (P<0.001) and sublethally (P<0.001) infected animals, suggesting that macrophages undergo cell death *in vivo* following *Shigella* ingestion. The number of neutrophils also significantly declined in lethally inoculated larvae (P<0.001), with an almost total neutrophil depletion by 24 hpi. In contrast, larvae controlling *Shigella* infection did not show a decrease in neutrophil number (P>0.05).

### A scavenger role for neutrophils during *Shigella* infection

To analyse in detail *Shigella*-phagocyte interactions *in vivo*, transgenic 3 dpf zebrafish larvae harboring red macrophages (*mpeg1*:G/U:mCherry) or red neutrophils (*lyz*:dsRed) were inoculated with sublethal doses of GFP-*Shigella*, and *Shigella*-phagocyte interactions were captured immediately thereafter using high resolution fluorescence confocal microscopy. Although only one of the two phagocyte populations was labelled in each of these experiments, non-labelled cells containing pooled GFP-*Shigella* could generally be assigned to the other phagocyte population in a non-ambiguous manner, based on their localization and motility [24], [26]. As soon as 20 min post infection (the earliest possible time point in our experimental setup), we observed that most blood borne bacteria were stuck on, or were already engulfed by, macrophages and few interacted with neutrophils (Fig. 3A, Video S1, data not shown), as previously observed using non-pathogenic *E. coli* [26] or *Listeria* [24]. However, unlike T3SS- *Shigella* (Fig. S3A, Video S2) or *E. coli* [26] which is degraded inside macrophages, we observed that wildtype *Shigella* were able to survive in macrophages and then the infected macrophages would burst (Fig. 3A, Fig. S3B, Video S1; see also Video S3). By contrast, neutrophils having phagocytosed similar amounts of *Shigella* quickly degraded the engulfed bacteria, as indicated by the diffuse GFP staining that transiently accumulated in these cells (Fig. 3B, Video S4; see also Fig. 3A, Video S1).

It is known that *in vitro* human macrophages infected by *Shigella* undergo cell death [41]. We also observed this phenomenon in zebrafish macrophages *in vivo* (Fig. 3C, Video S5; see also Video S4). To further investigate cell death induced upon *Shigella* infection, we performed TUNEL labeling of infected larvae and detected host cell DNA damage, a hallmark of cell death. Infected leukocytes were clearly labeled by TUNEL (Fig. S3C). Strikingly, we found that macrophages dead from *Shigella* infection were frequently engulfed by patrolling neutrophils, suggesting a newfound scavenger role for neutrophils during *Shigella* infection (Fig. 3D, Video S6; see also Video S4). In support of our live imaging studies, analysis by electron microscopy also highlighted that membrane and DNA from infected, dead cells are engulfed by leukocytes *in vivo* (Fig. S3D, S3E).

It is also well known that *Shigella* are able to actively invade non-immune cell types such as epithelial cells [42]. In zebrafish, invasion of some muscle fibers near the point of inoculation was observable from 5hpi (Fig. 3E, Video S7). We observed that *Shigella* were able to replicate within non-immune cells, which died after a few hours, releasing debris and bacteria that were quickly cleared by neutrophils (Fig. 3E, Video S7). We never observed bacterial invasion and/or replication *in vivo* with T3SS- *Shigella* (data not shown) nor *E. coli* (data not shown and [26]).

Collectively, these results demonstrate that, *in vivo*, *Shigella* is able to survive and replicate in macrophages and in some non-immune cells, ultimately leading to their death. Our results also highlight a scavenger role for neutrophils in engulfing and eliminating infected macrophages and non-immune cell types that have failed to control *Shigella* infection.

### *Shigella* escape to the cytosol and induce septin caging and autophagy in zebrafish larvae *in vivo*

We observed that in some cells (e.g., such as the macrophage or the muscle fiber displayed in Fig. 3D or 3E) *Shigella* seemed to occupy the entire cell (rather than a compartmentalized vacuole) strongly suggesting bacterial invasion of the cytosol. Because this cannot be fully ascertained from these live images, we fixed and labeled infected larvae for confocal microscopy to investigate the recruitment of septin cages to GFP-*Shigella*, a hallmark of cytosolic invasion [15]. In every larva infected (at 4 or 24 hpi), some GFP-*Shigella* were surrounded by SEPT7 cage-like structures. Confocal microscopy and deconvolution was used to obtain a high-resolution image of fluorescently-labeled assemblies and showed that septin structures assembled into cages around individual *Shigella* (Fig. 4A), in complete agreement with dimensions obtained from *Shigella*-septin cages measured in human cells [15]. Thus, *in vivo*, *Shigella* are able to invade the cytosol where they may be directed to autophagy by septin caging.

In mammalian cells, ATG8/LC3 is the marker widely used to define autophagosomes [43], [44]. Zebrafish Lc3 is an ATG8 homologue, and to confirm the targeting of bacteria to autophagy in zebrafish we infected transgenic GFP-Lc3 zebrafish larvae with *Shigella*. GFP-Lc3 was clearly recruited to intracellular *Shigella in vivo* (Fig. 4B, Video S8). Ultrastructural analysis of *Shigella*-infected zebrafish by electron microscopy confirmed the sequestration of cytosolic bacteria by autophagosomes (Fig. 4C, Video S9), classically observed as a double membrane structure [43], [44]. We also observed the *in vivo* recruitment of septin cages (Fig. S4A) and GFP-Lc3 (Fig. S4B, Video S10) to intracellular *M. marinum*, a natural fish pathogen previously shown *in vitro* to be recognized septin caging and autophagy [14], [15]. Taken together, the recruitment of autophagy markers to intracellular bacteria suggest the applicability of zebrafish larvae to study the manipulation of anti-bacterial autophagy.

### The autophagy receptor p62 is crucial for anti-*Shigella* defense *in vivo*

**Figure 5:**
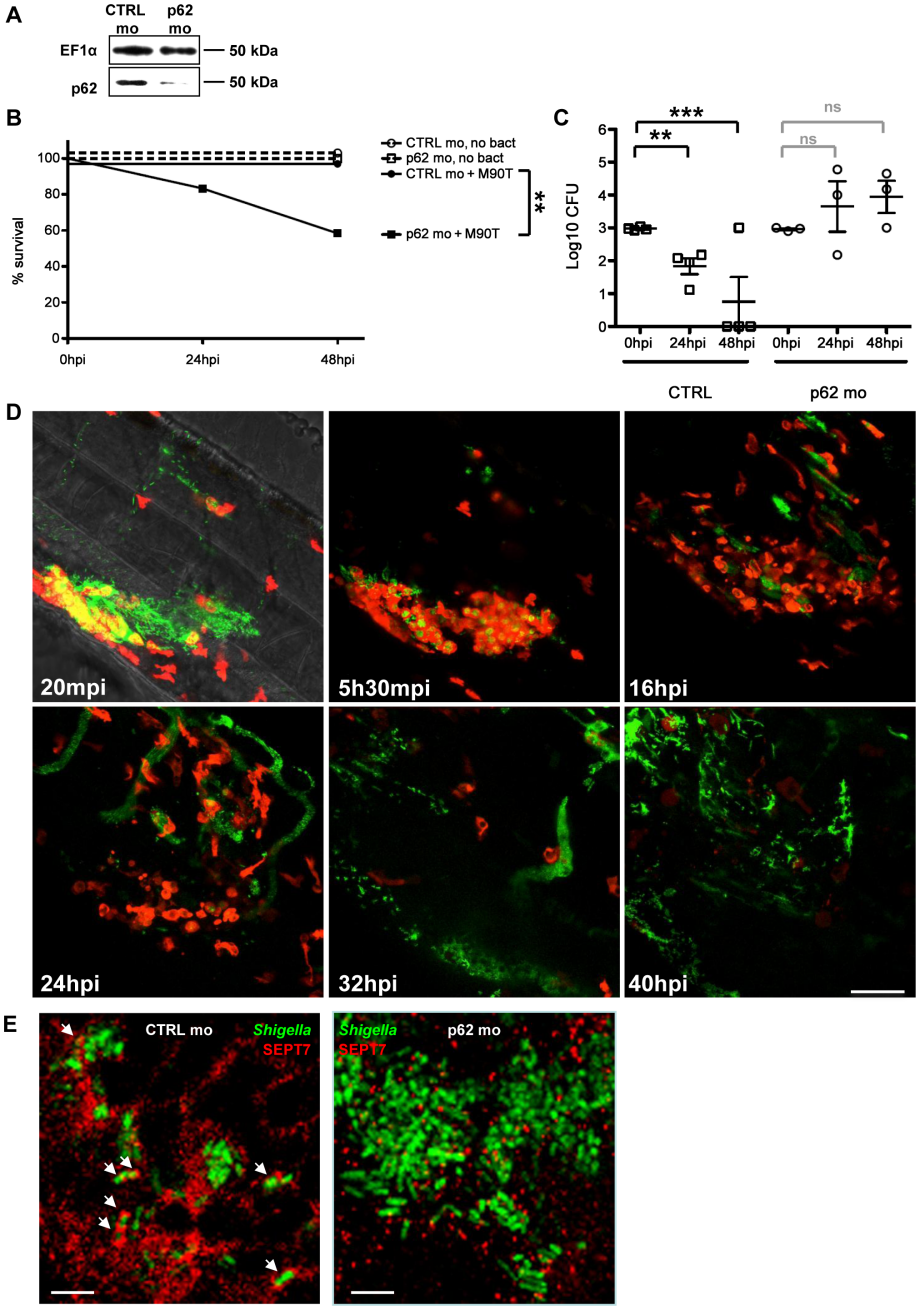
*In vivo* perturbation of bacterial autophagy via p62 depletion. A. Western blots of extracts of larvae injected with control (CTRL) or p62 morpholinos (mo), using antibodies against elongation factor 1-α (EF1α; control) or p62. Representative blots using lysates from AB fish; p62 depletion has also been observed in p62 morpholino-treated *mpeg*:G/U:mCherry and *lyz*:dsRed fish (data not shown). B. Survival curves of zebrafish larvae treated with control (CTRL) or p62 morpholinos and infected with a sublethal dose of *S. flexneri* (+M90T) or not infected (no bact). Representative experiment of at least 3 independent ones; n = 12 or more larvae per group. Significance testing performed by Log Rank test. **, P = 0.01. C. Bacterial counts in zebrafish larvae treated with control (CTRL) or p62 morpholinos and infected with a sublethal dose of *S. flexneri*. Enumeration of live bacteria in homogenates from individual larvae at various times postinfection measured by plating onto LB. CTRL larvae = open circles. p62-depleted larvae = open squares. One representative experiment of at least 3 independent ones; 3 larvae per condition. Of note, dead larvae at 24 or 48 hpi (overwhelmed with bacteria, as determined by fluorescence stereomicroscopy) were not included here, and only larvae having survived the infection (thus far) were included here (and in this case, statistics are in grey). Mean±SEM also shown (horizontal bars). Significance testing performed by Student's t test. ns, P>0.05; **, P<0.01; ***, P<0.001. D. Leukocyte behaviour and infection progression upon *Shigella* inoculation in p62 knockdown larvae over time. *lyz*:dsRed p62-depleted 3 dpf larva inoculated with GFP-*Shigella* and live imaged from 20 mpi to 40 hpi. Maximum intensity projection of 35 sections spaced by 2 µm. Frames extracted at various time points (20 mpi, 5h30mpi, 16 hpi, 24 hpi, 32 hpi and 40 hpi) suggest that neutrophils are unable to restrict bacterial proliferation. The infected larva is progressively depleted in neutrophils and overwhelmed with *Shigella*. Scale bar, 50 µm. E. Zebrafish larvae treated with either control (CTRL; left image) or p62 (right image) morpholinos were infected with GFP-*Shigella* for 4 (medium dose), fixed, labeled with antibodies against SEPT7 (red) and to GFP (green), and imaged by confocal microscopy. Arrows highlight some examples of *Shigella* entrapped in septin cages (CTRL) or not (p62 depleted) a 4 hpi. Scale bar, 5 µm.

Though autophagy is considered a crucial aspect of innate immunity to *Shigella*, this has not yet been tested *in vivo* [12], [13]. We proceeded to test the role of autophagy in the zebrafish response to *Shigella* infection. p62 is an autophagy receptor critical for autophagic recognition of bacteria *in vitro*, and is required for septin caging [14], [15]. Our analysis of the zebrafish genome revealed a single orthologue of *p62/sqstm1* (Fig. S5A). We thus designed a morpholino to knockdown p62 in zebrafish larvae (Fig. 5A). Fish depleted of p62 developed until 72 hpf with a survival rate similar to controls (∼80%), although we observed a slight developmental delay (data not shown). We then injected p62 morphants (i.e., zebrafish larvae injected with p62 morpholino oligonucleotides) with a sublethal inoculum of *Shigella* and observed that p62 knockdown significantly reduced host survival upon *Shigella* infection (P = 0.01; Fig. 5B). To understand the cause of increased death in p62 morphants, we examined the bacterial burden in these fish. CFU counts clearly showed that p62 knockdown during *Shigella* infection prevents bacterial clearance (Fig. 5C).

T3SS- *Shigella* and *E. coli* may not escape to the cytosol of cells that have engulfed it, and are not expected to be targeted to autophagic degradation *in vivo*. Thus, as a control, we infected p62 morphants with T3SS- *Shigella* or *E. coli*. In contrast to infection using wildtype *Shigella*, we observed that the knockdown of p62 had no impact on growth of T3SS- *Shigella* nor *E. coli*, nor did it impact zebrafish survival (Fig. S5B–S5E). These data suggest that p62-mediated autophagy is critical for the control of bacteria able to escape to the cytosol.

We tested whether the inflammatory cytokine response to *Shigella* was modulated by p62 knockdown. Experiments suggested that the inflammatory response was stronger in p62 morphants inoculated with *Shigella* (data not shown), yet these results may reflect the increased bacterial burden of p62 morphants compared to controls. Using confocal microscopy, we observed that in the absence of p62, initial bacterial engulfment by phagocytes (macrophages and neutrophils) was not affected; yet uncontrolled *Shigella* proliferation ensued, accompanied by the ultimate lysis of leukocytes and death of the infected larvae (Fig. 5D). These observations indicated that, as compared to control fish, p62 morphants are not able to restrict *Shigella* proliferation and, as a result, they are more susceptible to *Shigella* infection. We evaluated septin caging in p62 morphants. In every larva infected (at 4 or 24 hpi), septin recruitment to *Shigella* was clearly reduced (Fig. 5E), in agreement with previous *in vitro* work showing that septin cage assembly is more efficient once the process of autophagy has been initiated [14], [15]. Together, these results strongly suggest that p62-mediated autophagy is a critical component of anti-*Shigella* defense *in vivo*.

### Autophagy stimulation by rapamycin does not protect fish from *Shigella* infection

Rapamycin is an inhibitor of mTOR, a serine/threonine protein kinase that regulates a wide range of cellular responses and is a suppressor of autophagy [45]. Autophagy is classically activated *in vitro* by treating cells with rapamycin, and in agreement with previous work [46], [47], we observed that critical autophagy components in zebrafish larvae can respond to rapamycin, i.e., p62 is degraded and LC3-II accumulates (data not shown). Importantly, we saw no effect of rapamycin (at the dose we applied throughout this study) on the development of uninfected larvae, in agreement with a previously published study showing that rapamycin has no discernible effect on zebrafish development past 76 hpf [48].

**Figure 6:**
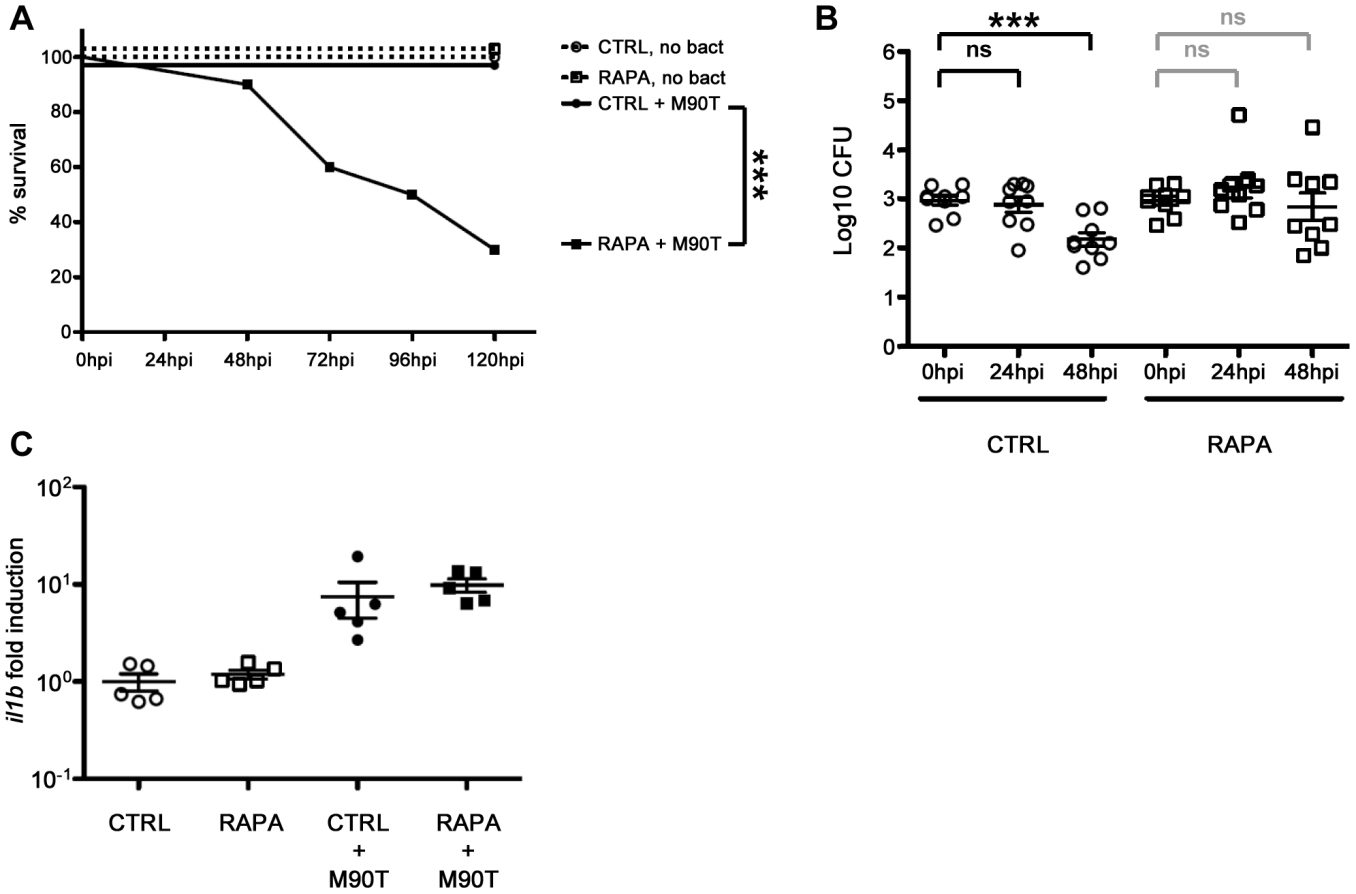
*In vivo* perturbation of autophagy via rapamycin stimulation. A. Survival curves of zebrafish larvae treated with control (CTRL) or rapamycin (RAPA) and infected with a sublethal dose of *S. flexneri* (+M90T) or not infected (no bact). Representative experiment of at least 3 independent ones; n = 10 or more larvae per group. Significance testing performed by Log Rank test. ***, P = 0.001. B. Bacterial counts in zebrafish larvae treated with control (CTRL) or rapamycin (RAPA) and infected with a sublethal dose of *S. flexneri*. Enumeration of live bacteria in homogenates from individual larvae at various times postinfection measured by plating onto LB. CTRL larvae = open circles. RAPA-treated larvae = open squares. n = 3 larvae per treatment, pooling of 3 independent experiments. Of note, dead larvae at 24 or 48 hpi (overwhelmed with bacteria, as determined by fluorescence stereomicroscopy) were not included here, and only larvae having survived the infection (thus far) were included here (and in this case, statistics are in grey). Mean±SEM also shown (horizontal bars). Significance testing performed by Student's t test. ns, P>0.05; ***, P<0.001. C. Inflammatory response is not affected in rapamycin-treated larvae. Expression of *il1b* transcripts at 96 hpf in larvae injected with ∼10^3^ *S. flexneri* at 72 hpf (+M90T) or in uninfected controls, treated after infection with rapamycin (RAPA) or vehicle only (DMSO, i.e., CTRL). *Il1b*/*ef1α* transcript number ratios, normalized to the mean value in uninfected controls. Results for 5 pools of 3 larvae per group, mean±SEM also shown (horizontal bars).

The stimulation of autophagy by rapamycin is generally viewed as helping to clear bacterial infection [2], [49]. To test this *in vivo*, we infected zebrafish larvae with a sublethal dose of *Shigella* and immediately after infection, treated these fish with rapamycin. Surprisingly, rapamycin treatment in zebrafish during *Shigella* infection significantly reduced host survival (P = 0.001; Fig. 6A), albeit at a slower rate than observed for p62 morphants (Fig. 5B). To understand the cause of increased death due to rapamycin treatment, we examined the bacterial burden in these fish. CFU counts showed that rapamycin treatment in zebrafish during *Shigella* infection may result in more bacterial growth (Fig. 6B). Using fluorescence stereomicroscopy, we confirmed that dying larvae were overwhelmed with GFP-*Shigella* (data not shown). We tested if an impairment of the inflammatory response could explain the enhanced susceptibility of rapamycin-treated fish; however, *il1b* induction was not found to be modulated by rapamycin *in vivo* (Fig. 6C). Moreover, rapamycin treatment of the T3SS- *Shigella*- or *E. coli*-infected larvae did not affect zebrafish survival nor bacterial load (Fig. S6A–S6D), indicating that rapamycin-treated fish are still able to mount an innate immune response.

Taken together, these results show that rapamycin treatment of bacterial infection can lead to harmful effects *in vivo*, unlike what may be expected from studies performed *in vitro*. This suggests possible difficulties in therapeutic modulation of autophagy to resolve bacterial infection, until more specific activators of autophagy are discovered.

## Discussion

We describe here the successful establishment of a new *S. flexneri* infection model system, the larval zebrafish, which allows a detailed real-time analysis of host-pathogen interactions *in vivo*. Similar to other bacteria such as *L. monocytogenes* [24], *Bacillus subtilis* [23], *S.* Typhimurium [50] and *Staphylococcus aureus* [31], both *S. flexneri* and *E. coli* are quickly cleared from the bloodstream by phagocytes, notably macrophages to which they readily stick. However, while T3SS- *Shigella* or *E. coli* are rapidly degraded by phagocytes [26], we found that *Shigella* may persist and replicate inside macrophages, eventually killing them; by contrast, neutrophils that have engulfed similar amounts of *Shigella* efficiently kill the bacteria. We also observed that *Shigella* is able to invade and replicate inside non-immune cells, and cytosolic invasion of *Shigella* was evident from the formation of septin cages and Lc3-positive autophagosomes. Phagosome escape is known to be critical for the virulence of *L. monocytogenes* and *M. marinum* in the zebrafish [24], [25], and, as shown here, seems also critical for the virulence of *S. flexneri* in the zebrafish.

The zebrafish model of *S. flexneri* infection displays several interesting features. We show that pathogenesis of *Shigella* in zebrafish larvae *in vivo* is strictly dependent upon its T3SS. Previous work has shown that *Shigella* virulence factors may be expressed at temperatures lower than 37°C [51], and in agreement with this, we detected the expression of *icsA* and *icsB* in zebrafish larvae infected at 28°C. We have also shown that the zebrafish larva constitutes a relevant model of human infection as regards to host-phagocyte interactions. Strikingly, the numbers of both macrophages and neutrophils dramatically decreased in larvae unable to control *Shigella* proliferation, and leukocyte depletion was associated with bacteremia preceding the death of the larvae. Neutrophil depletion has also been observed upon infection of zebrafish larvae with *Salmonella* [50] and *Staphylococcus* [52], upon infection of mice with *Listeria* [53], and may emerge to be a critical correlate of bacterial overgrowth. In humans neutropoenia is clinically predictive of failure to resolve infection, and is associated with bacteraemia that leads to a worse prognosis. The control of infection by neutrophils will deserve careful attention in future studies.

Pioneering studies performed *in vitro* suggested that *Shigella* may induce apoptosis in human macrophages [41]. More recent work has shown that *Shigella*-infected macrophages undergo caspase-1-mediated cell death, termed pyroptosis, which is a pathway of programmed cell death associated with an inflammatory response [54]. We have characterized cell death during *Shigella* infection *in vivo*. By live imaging we have followed the fate of individual leukocytes infected with *Shigella* in real time (e.g., Video S1, S3, S5). By *in situ* analysis of fixed larvae, we have clarified that some infected leukocytes are dying based on labelling of DNA strand breaks (TUNEL; Fig. S3C). Using EM, we have observed that infected leukocytes may undergo morphological changes associated with pyroptosis, e.g., obvious plasma membrane rupture (Fig. S3B). Macrophages dead from *Shigella* infection were frequently engulfed by patrolling neutrophils, suggesting an unexpected role for neutrophils during *Shigella* infection. This is comparable to a recent report for *M. marinum* [55]. Moreover, our live imaging studies have revealed a new scavenger role for neutrophils in clearing cell debris and live released bacteria from dying infected cells *in vivo*. The zebrafish model will be particularly useful to investigate these different host cell responses to infection, as well as to dissect the role of implicated *Shigella* effectors, *in vivo*.

Autophagy is recognized as a crucial defense mechanism against intracytosolic bacteria [2], [12], [13]. In mammalian cells *in vitro*, virulent *S. flexneri* bacteria escape from the phagosome and invade the cytosol of cells where they are recognized by septin cages and autophagy [14], [15]. Yet our understanding of autophagy during bacterial infection would benefit from an experimental model in which the sequence of host-pathogen interactions resulting in autophagy can be validated and dissected *in vivo*. The optical accessibility of zebrafish larvae allowed us to image *Shigella*-septin caging in the infected organism, an achievement that has never before been accomplished using mammalian host models. Septin recruitment to intracytosolic *Shigella* was consistently detectable in infected larva, and promises to be an exciting tool to investigate the role of the cytoskeleton in autophagy *in vivo*. To complement evidence that septin cages entrap bacteria targeted to autophagy *in vivo*, we have shown *in vivo* recruitment of GFP-Lc3 to *Shigella* by confocal microscopy (Fig. 4B for analysis of fixed samples and Video S8 for live imaging). In addition, we have obtained ultrastructural analysis of *Shigella* autophagosomes by EM (Fig. 4C), and clearly show the cytosolic sequestration of *Shigella in vivo* by double membrane vacuoles. By confocal microscopy (Fig. S4A for analysis of fixed samples and Video S10 for live imaging), we have shown *in vivo* recruitment of septin caging and GFP-Lc3 to *M. marinum*, a natural fish pathogen previously shown to be recognized septin caging and autophagy *in vitro*. All of these observations fully validate the zebrafish as a new model for the *in vivo* study of bacterial autophagy.

p62 is a well-characterized autophagy receptor and is increasingly recognized as a critical component of innate immunity (reviewed in [12], [13]). In agreement with this, we show that p62-mediated bacterial autophagy is crucial to control bacterial pathogenesis *in vivo*. On the other hand, we also observed that rapamycin, while stimulating autophagy as expected [46], [47], may increase bacterial replication and decrease zebrafish survival. Interestingly, *in vitro* treatment of Hela cells with rapamycin for 1–4 hrs was observed to have no significant effect on *Shigella* [56]. In our model, leaving zebrafish larvae in rapamycin for longer periods of time (e.g., 5 days) may lead to harmful effects *in vivo*; this that will require attention in future studies. In agreement with data obtained using *Shigella* (Fig. 6A, 6B), we have observed that rapamycin treatment of *Listeria*-infected zebrafish may not promote host survival or bacterial clearance (Fig. S6E, S6F). This effect of rapamycin could be due to processes other than autophagy, for the rapamycin target mTOR affects many other cellular processes [45], yet these data clearly serve as an important warning for the therapeutic implications of rapamycin treatment. Overall, these data strongly suggest that zebrafish survival depends on the appropriate autophagic response to control intracellular bacterial infection.

To conclude, we show that the zebrafish larva represents a valuable new host for the analysis of *S. flexneri* infection. Interactions between bacteria and host phagocytes can be imaged at high resolution *in vivo*, and the zebrafish model should prove useful for understanding the cell biology of *Shigella* infection. It may become possible in the future to observe *S. flexneri* phagosome escape, actin tail formation and septin caging *in vivo* in real time by developing new transgenic zebrafish lines. Here, we use zebrafish larvae to investigate the role of bacterial autophagy in host defense at the whole organism, cellular and single-cell level, and observed that the perturbation of autophagy can adversely affect host survival in response to *Shigella* infection. Future work will visualize and characterize the molecular determinants of autophagy during microbe-cell interactions *in vivo*, using several different bacterial and viral pathogens.

## Material and Methods

### Ethics statement

Animal experiments conducted at the Institut Pasteur were performed according to European Union guidelines for handling of laboratory animals (http://ec.europa.eu/environment/chemicals/lab_animals/home_en.htm) and were approved by the Institut Pasteur Animal Care and Use Committee and the Direction Sanitaire et Vétérinaire de Paris under permit #A-75-1061. Animal experiments performed at Imperial College were performed according to the Animals (Scientific Procedures) Act 1986, and were fully approved by the Home Office (Project license: PPL 70/7446).

### Zebrafish care and maintenance

Wild-type AB purchased from the Zebrafish International Resource Center (Eugene, OR), and the Tg(*lyz*:dsRed)*^nz50^*, Tg(*mpeg1*:Gal4FF)*^gl25^*, Tg(*mpeg1*:Gal4FF^gl25^/UAS:kaede), Tg(UAS-E1b:nfsB.mCherry)*^c264^*, and GFP-Lc3 transgenic zebrafish lines have been previously described [46], [57]–[59]. Eggs were obtained by marble-induced spawning, bleached according to protocols described in The zebrafish book [60], and then kept in Petri dishes containing Volvic source water supplemented with 0.3 µg/ml of methylene blue and, from 24 hours post fertilization (hpf) onwards with 0.003% 1-phenyl-2-thiourea (Sigma-Aldrich) to prevent melanin synthesis. Embryos were reared at 28°C or 24°C according to the desired speed of development; infected larvae were always kept at 28°C. All timings in the text refer to the developmental stage at the reference temperature of 28.5°C [61]. Larvae were anesthetized with 200 µg/ml tricaine (Sigma-Aldrich) during the injection procedure as well as during the *in vivo* imaging.

### Zebrafish fibroblast-like cell line establishment

Fibroblast-like cells were derived from AB zebrafish as follows. After a zebrafish (AB strain) has been sacrificed by overexposure to eugenol, a dorsal slice comprising skin, muscle and the dorsal fin was taken and trypsinised under constant mild shaking for 5 min. The supernatant was collected in modified MacPherson Stoker Eagle's medium (Eurobio) supplemented with 10% fetal calf serum (FCS), 100 IU/mL penicillin and 100 mg/mL streptomycin. The cell suspension was centrifuged for 5 min at 1000 g, and cells were resuspended in culture medium. The rainbow trout fibroblast cell line RTG-2 was then used as feeder, and zebrafish cells were grown on a monolayer of RTG2 for almost one year. At each passage, the co-culture was kept for 1–2/3 weeks at 20°C then put for a few days at 30°C; at this temperature, trout cells died and the remaining zebrafish cells used to seed the next passage over a new RTG-2 monolayer. After one year, ZF-AB cells started to be able to grow without feeder. Once established, fibroblast-like cells derived from AB zebrafish (ZF-AB) cells were cultured in minimum essential medium plus GlutaMAX (Invitrogen) supplemented with 1 mM sodium pyruvate (Invitrogen), 0.1 mM nonessential amino acid solution (Invitrogen), and 10% FCS. Zebrafish cells were grown at 28°C.

### Infection of cell lines and microscopy

1–1.5×10^5^ ZF-AB or HeLa cells were plated on glass coverslips in 6-well plates (Techno Plastic Products) and used for experiments 48 h later. Cells on coverslips were fixed for 15 min in 4% paraformaldehyde and then washed with 1× PBS and processed for immunofluorescence (IF). After 10 min of incubation in 50 mm ammonium chloride, cells were permeabilized for 4 min with 0.1% Triton X-100 and then incubated in 1× PBS. Incubation with primary or secondary antibodies was performed in 1× PBS. Vectashield hard set mounting medium with DAPI (Vector Laboratories) or mounting medium for IF (Interchim) was used.

*Shigella* was added to cells using 400 µl of growth (A_600 nm_ = 0.6) or using 200 µl of overnight growth (A_600 nm_ = 0.6) from single colony, and was diluted in minimum essential medium and added directly to cells for imaging analyses. Bacteria and cells were centrifuged at 700× g for 10 min at 21°C and then placed at 28°C for 30 min, washed with minimum essential medium, and incubated with fresh gentamicin-containing complete medium (50 µg/ml) for 4 h, after which they were washed with 1× PBS and fixed and processed for IF.

Images were acquired on a fluorescence inverted microscope Axiovert 200 M (Carl Zeiss MicroImaging, Inc.) equipped with a cooled digital charge-coupled device camera (Cool SNAPHQ, Photometrics) driven by Metamorph Imaging System software (Universal Imaging Corp).

### Bacteria and bacterial infections

Bacterial strains used in this study were wild-type invasive of *S. flexneri* serotype 5a (M90T; BUG 2505) [15], M90T expressing green fluorescent protein (GFP) (GFP-*Shigella*; BUG 1908) [15], dsRed [62] or mCherry (pFPV 25.1 mCherry construct), T3SS− noninvasive variant (*ΔmxiD*) expressing dsRed [62], *E. coli* K12 bacteria expressing dsRed [26], and *M. marinum* M strain expressing GFP or dsRed [15]. In the case of *E. coli*, infections were performed as previously described [24], [26]. *S. flexneri* were cultured overnight in trypticase soy, diluted 80× in fresh trypticase soy, and cultured until A_600 nm_ = 0.6. *M. marinum* M-GFP and M-DsRed were cultured at 30°C in Middlebrook 7H9 (BD Biosciences) supplemented with 0.2% glycerol, 0.05% Tween 80 and 10% ADC Enrichment (Fisher Scientific), diluted 48 h prior to infection in fresh media, and cultured until OD600 nm = 0.6. For injection of zebrafish larvae, bacteria were recovered by centrifugation, washed, resuspended at the desired concentration in PBS. In the case of *M. marinum*, bacteria were also passaged through a 26 gauge needle to dissociate bacterial clumps and homogenize the bacteria prior to infection. 3 day post-fertilisation (dpf) anesthetized zebrafish larvae were microinjected intravenously (iv) with 0.5–2 nl of bacterial suspension as described previously [24]. The exact inoculum was checked a posteriori by injection in a water drop and plating onto LB agar. Infected larvae were transferred into individual wells (containing 1 ml of Volvic water in 24-well culture plates), incubated at 28°C and regularly observed under a stereomicroscope. Infections with a quantified standard dose were performed at least 3 times per strain of bacteria. Survival curves with graded doses as depicted were repeated at least 3 times.

### Measurement of bacterial burden

At the indicated times, animals were anesthetized, rinsed, and collected in 30 µl of sterile water. The animals were lysed in 200 µl of 0.4% Triton X-100 and homogenized through a 26-gauge needle (five up-and-down sequences). Serial dilutions of the homogenates were plated onto LB agar, and CFU were enumerated after 24 h of incubation at 37°C; only colonies with the appropriate morphology and color were scored.

### Microscopy of zebrafish (infected or not)

Anesthesized zebrafish larvae were fixed for 2 h at RT or overnight at 4°C in 4% paraformaldehyde with 0.4% triton, then washed with PBS 0.1% tween, and processed for IF. In brief, after 20 additional minutes of PBS 1% triton, larvae were washed 3×5 minutes in PBS 0.4% triton, then incubated in blocking solution: PBS 1× supplemented with 10% sheep serum, 1% DMSO, and 0.1% tween, for 1 hour. Primary antibodies were diluted in blocking solution and were applied to larvae overnight at 4°C. To remove primary antibodies, larvae were washed 4×15 min in PBS +0.1% tween. After washes, larvae were placed in blocking solution for 1 h at RT. Secondary antibodies were diluted in block solution and were applied to larvae overnight at 4°C. To remove secondary antibodies, larvae were washed 4×15 min in PBS 0.1% Tween. Fluorescently labeled larvae were then cleared by progressive transfer to 80% glycerol.

Quantification of macrophages and neutrophils numbers on fixed and labeled transgenic reporter larvae was performed as following. Briefly, brightfield, dsRed and GFP images of whole fixed larvae were taken using a Leica Macrofluo Z16 APOA (zoom 16∶1) equipped with a Leica PlanApo 2.0× lens, and a Photometrics CoolSNAP *HQ* ^2^ camera. Images were captured using the Metavue software version 7.5.6.0 (MDS Analytical Technologies). Then, pictures were analyzed and positive cells for red fluorescence counted using the ImageJ software version 10.2 (developed by the National Institute of Health). Neutrophils numbers were obtained with *lyz*:dsRed larvae, macrophage numbers with *mpeg1*:G/U:mCherry larvae. Counts shown in Fig. 2B are numbers of leukocytes per image.

Examination of septin recruitment *in vivo* was performed using a confocal microscope (Zeiss LSM510). Larvae were infected with GFP-*Shigella* in the mesenchyme near the caudal vein or in the hindbrain, and fixed and labeled for microscopy 4 or 24 hpi. Z-stacks of infected larvae were acquired for each sample using a 63× or 100× objective and a slice increment of 0.2 µm. These stacks were then deconvoluted (Huygens software) and analysed using ImageJ according to previously established criteria [14], [15], [63].

### Live imaging, image processing and analysis

To perform whole-body *in vivo* imaging, anaesthetized zebrafish larvae were oriented and immobilized in 1% low melting point agarose in 60 mm plastic bottom Petri dishes (up to 9 fish mounted and imaged per dish), then covered with 2 ml Volvic water containing tricaine as described previously [26]. Transmission and fluorescence widefield imaging was done using a Nikon Biostation IM–Q S2 equipped with a DS-Qi camera. Imaging was typically performed at 26°C with a 10× (NA 0,5) dry objective. Multiple-field *Z*-stacks with a 10 µm *Z*-step were acquired every 30 minutes.

To perform high resolution confocal live imaging, injected larvae were positioned in 35 mm glass-bottom dishes (Inagaki-Iwaki). To immobilise the larva in the dish a 1% low-melting-point agarose solution covering the entire larva was used. The immobilised larvae were then covered with 2 ml Volvic water containing tricaine. Confocal microscopy was performed at 23–26°C using a Leica SPE inverted microscope and a 40× oil immersion objective (ACS APO 40×1.15 UV) as previously described [26]. A Leica SP8 confocal microscope equipped with two PMT and an Hybrid detector (HyD) and a 20× oil immersion objective (HC PL APO CS2 20×/0.75) was used to live image infected larvae (represented in Figure S4B, Video S8, Video S10).The 4D files generated by the time-lapse acquisitions were processed, cropped, analysed and annotated using the LAS-AF Leica software. Acquired Z-stacks were projected using maximum intensity projection and exported as AVI files. Frames were captured from the AVI files and handled with Photoshop software to mount figures. AVI files were also cropped and annotated with ImageJ software, then compressed and converted into QuickTime movies with the QuickTime Pro software.

### Electron microscopy

For standard ultrastructure analyses, as described in [24], [64], anesthetized embryos were fixed in 0.5% glutaraldehyde in 200 mM sodium cacodylate buffer for 2 h, washed in buffer and secondarily fixed in reduced 1% osmium tetroxide, 1.5% potassium ferricyanide for 60 min. The samples were washed in distilled water and stained overnight at 4°C in 0.5% magnesium uranyl acetate, washed in distilled water and dehydrated in graded ethanol, infiltrated with propylene oxide and then graded Epon/PO mixtures until final embedding in full Epon resin in coffin moulds (allowing different orientations) and polymerised at 56°C overnight. Semi-thin survey sections were cut and stained and final ultrathin sections (typically 50–70 nm) and serial sections were collected on Formvar coated slot grids and stained with Reynold's lead citrate and examined in a FEI Tecnai electron microscope with CCD camera image acquisition.

### qRT-PCR

RNA was extracted from snap-frozen larvae using Trizol (Invitrogen). cDNA was obtained using M-MLV H- reverse-transcriptase (Promega) with a dT17 primer. Quantitative PCR was then performed on an ABI7300 thermocycler (Applied Biosystems) using SYBR green reaction power mix (Applied Biosystems). The following pairs of primers were used: EF1α (GCTGATCGTTGGAGTCAACA and ACAGACTTGACCTCAGTGGT); IL1b (GAGACAGACGGTGCTGTTTA and GTAAGACGGCACTGAATCCA). Quantifications were performed on triplicate wells, and taking into account the previously measured yield of the reaction as described in [65]. To normalize cDNA amounts, we used the housekeeping gene EF1α transcripts [66].

### *icsA, icsB* expression *in vivo*

*S. flexneri* M90T was injected into the hindbrain ventricle of zebrafish larvae and incubated at 28°C for 4 h (high dose), 12 h (medium dose) and 24 h (medium dose). RNA was extracted from 10 snap-frozen larvae per timepoint using Trizol (Invitrogen). cDNA was obtained using QuantiTect Reverse Transcription Kit (Qiagen). PCR was then performed using a PTC-225 Poltier Thermal Cycler (MJ Research) and RedTaq reaction mix (Applied Biosystems). The following pairs of primers were used: *icsA* (AATCAATAAGGGCACGTTCG and TCGCCATCTGTATCATTCCA); *icsB* (GCATCGGTACAGCCAAAAAT and GTATGAGTGGCAAGCGTTGA).

### Antibodies

Rabbit polyclonal antibodies used were anti-SEPT7 (R170) [15], anti-p62 (Cliniscience, PM045), anti-dsRed (Clontech Laboratoires), anti-elongation factor 1-α (EF1α; GeneTex); to label GFP chicken polyclonal and mouse monoclonal antibodies anti-GFP (Abcam) were used. Secondary antibodies used were Cy3-, Cy5- (Jackson ImmunoResearch Laboratories), Alexa Fluor 488-, or Alexa Fluor 546-conjugated goat anti-rabbit, anti-chicken or goat anti-mouse (Molecular Probes). F-actin was labeled with Alexa Fluor 488-, 546-, or 647-phalloidin (Molecular Probes). Whole-mount immunohistochemistry of infected zebrafish cells was performed using standard protocols [14], [15]. For immunoblotting, total cellular extracts were blotted with the above-mentioned antibodies followed by peroxidase-conjugated goat anti-mouse or anti-rabbit antibodies (Biosys Laboratories). Anti-EF1α was used throughout as a loading control. Proteins were run on 8, 10 or 14% acrylamide gels.

### Morpholinos and pharmacological inhibitors

Antisense morpholino oligonucleotides were obtained from GeneTools (www.gene-tools.com). After thawing, morpholinos are heated at 65°C for 10 min to ensure complete dissolution, and diluted to the desired concentration (typically, 250 to 500 µM, for a total injected amount of 4 ng) in morpholino buffer (120 mM KCl, 10 mM Hepes pH 7.2) containing 0.1% phenol red. Injections were performed into 1-cell embryos at 1 nL per embryo. Control (TACCAAAAGCTCTCTTATCGAGGGA, with no known target on the zebrafish genome) and p62-specific (CACTGTCATCGACATCGTAGCGGAA, targeting the start AUG codon) morpholinos were used.

For rapamycin treatment, protocols were adapted from [46]. Larvae were treated with rapamycin (Calbiochem) for 12 h (50 nM), and treatment was continued throughout infection.

### Statistical analysis

Significance testing for performed by Log Rank test (survival curves), Student's t test (on log values of CFU counts), or by ANOVA with Bonferroni posttest (leukocyte counts). The level of significance is shown as follows: ns, P>0.05; *, P<0.05; **, P<0.01; ***, P<0.001.

## Supporting Information

Figure S1 **Characterization of zebrafish septin 7 and** ***Shigella*** **virulence factors.** A. Alignment of zebrafish and human septin 7 proteins. Human septin 7 (NM_001788.5) and its three zebrafish orthologues: sept7a (deduced from a sequence we re-amplified and cloned because of inconsistencies in the available sequences and deposited at GenBank KC513820, gene ENSDARG00000052673 on chromosome 16), sept7b (NP_001071211, gene ENSDARG00000019649 on chromosome 25) and sept7c (NP_001242958, gene located on chromosome 19 in a region improperly assembled on zv9, broken in three parts: ENSDART00000126747, ENSDARG00000075198 and ENSDART00000131014). B. Western blot of larval extracts using antibodies against SEPT7 as described in Fig. 1A. Shown here is complete lane of SEPT7 blot and antibody specificity. C. *Shigella* forms actin tails and recruits septin cages in HeLa cells at 28°C. Immunofluoresence microscopy of HeLa cells infected with *Shigella* for 4h40min at 28°C. F-actin (green), SEPT7 (red), DAPI (blue). Note septin cage-like structures surrounding DAPI-labeled *Shigella* (arrows). Scale bar, 2 µm. D. Survival curves of 72 hpf larvae injected with various doses of T3SS- *S. flexneri* and incubated at 28°C for 48 hpi. The effective inoculum, quantified a posteriori, was classified as low (<10^3^ CFU, open circles; effective range: 0.6–11.7×10^2^ CFU), medium (∼4×10^3^ CFU, open triangles; effective range: 1.2–6.0×10^3^ CFU) or high (∼10^4^ CFU, open squares; effective range: 6.1–33.3×10^3^ CFU). Mean±SEM of 24 larvae from 2 independent experiments per inoculum class. E. Enumeration of live bacteria (T3SS- *Shigella*) in homogenates from individual larvae at various times post infection measured by plating onto LB. Low dose inoculum = open circles. Medium or high dose inoculum = open squares. Mean±SEM also shown (horizontal bars). Significance testing performed by Student's t test. *, P<0.05; **, P<0.01; ***, P<0.001. F. *icsA* and *icsB* expression *in vivo*. *S. flexneri* M90T was injected into the hindbrain ventricle of zebrafish larvae and were incubated at 28°C for 4 h (high dose), 12 h (medium dose) or 24 h (medium dose). RNA was extracted from 10 larvae per timepoint and converted into cDNA using reverse transcriptase (+RT) or not (−RT), PCR amplified, and amplicons were run on 1% agarose gel. Genomic DNA from *Shigella* was used as a positive control.
(TIF)

Figure S2 ***In vivo*** **macrophage and neutrophil behaviours and progression of the infection over time upon sublethal and lethal** ***Shigella*** **inocula.** Images of the caudal area of *Mpeg1*:G/U:mCherry (red macrophages, upper panel) or *lyz*:dsRed (red neutrophils, lower panel) 3 dpf larvae iv injected with sublethal and lethal GFP-*Shigella* inocula. Live imaging by widefield microscopy, overlay of red and green fluorescence. Images at 2, 12 and 24 hpi are shown. (Bottom) Scheme of a 72 hpf larva: the imaged region is boxed, and arrow shows the injection site.
(TIF)

Figure S3 **Characterisation of** ***Shigella*** **-dependent macrophage death.** A. Frames extracted from *in vivo* time-lapse confocal imaging sessions of 3 dpf larvae injected in the bloodstream with GFP-*Shigella* (3000–4000 bacteria) and T3SS- DsRed *Shigella* (6000–8000 bacteria). Caudal area, rostral to bottom right, dorsal to top right. Maximum intensity projection from 26 planes every 2 µm. Scale bar, 20 µm. Upper panel: *mpeg1*:G/U:mCherry larva (red macrophages); first frame at 20 mpi. By 20 mpi, GFP-*Shigella* have already adhered to or have been engulfed by macrophages (red cells, white arrows, number 1 to 5). Tracked infected macrophages are killed by GFP-*Shigella*; they progressively round up and burst, releasing in the extracellular milieu live GFP-*Shigella*. Note that at the end of the sequence (about 3 hpi) the bacteria are still in the blood. See also Video S3. Lower panel: 72 hpf Tg(*mpeg1*:Gal4FF^gl25^/UAS:kaede) larva (green macrophages); first frame at 20 mpi. By 20 mpi T3SS- DsRed-*Shigell*a have been engulfed by macrophages (white arrow, number 1 to 6). Tracked infected macrophages progressively kill the engulfed bacteria (diffuse red staining accumulating in their phagosomes) over time. Note that at the end of the sequence (about 3 hpi) all the red bacteria have been cleared from the blood, without any sign of cell death. See also Video S2. B. Bacteria are killing host cells (leukocytes) in the caudal vein. From the ultrastructure of the infected dying cell, plasma membrane rupture and damaged cellular material are observed, hallmarks of pyroptosis. Zebrafish larvae were infected in the tail muscle with GFP-*Shigella* (medium dose) for 4 h and fixed for EM. Shown here are 8 serial sections in a part of infected larvae. The first section shows the plasma membrane is ruptured (black arrow) and the cellular material is damaged. In the remaining sections, a bacterium (labeled in frame 5 as M90T) is inside the damaged cell. Scale bar, 0.5 µm. C. Infected cells are dying as shown by TUNEL labeling. Zebrafish larvae were infected in the tail muscle with GFP-*Shigella* for 4 (medium dose), fixed, labeled with antibodies against GFP (green), labeled for TUNEL, and imaged by confocal microscopy. Scale bar, 1.5 µm. D. A leukocyte with engulfed bacteria and dead cell. Zebrafish larvae were infected in the tail muscle with GFP-*Shigella* (medium dose) for 4 h and fixed for EM. n = leukocyte nucleus; Boxed region = DNA condensation and cellular membrane debris from a dead cell altogether within a phagosome. Scale bar, 2 µm. E. A leukocyte with engulfed bacteria and dead cell. Zebrafish larvae were infected in the tail muscle with GFP-*Shigella* (medium dose) for 4 h and fixed for EM. E2 is an expanded region of boxed region in E1, E3 is an expanded region of boxed region in E2 (i.e., DNA condensation and cellular membrane debris from a dead cell altogether within a phagosome). n = leukocyte nucleus. Scale bar, 5 µm (E1), 2 µm (E2) or 0.5 µm (E3).
(TIF)

Figure S4 ***M. marinum*** **escape to the cytosol and induce septin caging and autophagy in zebrafish larvae** ***in vivo*** **.** A. Zebrafish larvae were infected subcutaneously with GFP-*M. marinum* for 48 h (low dose), fixed, labeled with antibodies against SEPT7 (red) and to GFP (green) and imaged by confocal microscopy. Scale bar, 5 µm. B. GFP-Lc3 zebrafish larvae were infected subcutaneously with DsRed-*M. marinum* and live imaged by confocal microscopy. Shown here is an example of 3 GFP-Lc3 positive leukocytes having engulfed *M. marinum* and highlights Lc3 recruitment to bacteria. The maximum intensity projection from 5 planes every 2 µm is shown. Scale bar, 10 µm. See also Video S10.
(TIF)

Figure S5 **Infection of p62-depleted larvae.** A. Alignment of zebrafish and human p62. Alignment of human p62/sqstm1 (NP_003891) and its zebrafish orthologue (gene ENSDARG00000075014 on chromosome 14; cDNA sequence deduced from EST consensus and deposited at GenBank KC513821). Analysis by Ensembl Genetree shows p62/sqstm1 is highly conserved between human and zebrafish: http://www.ensembl.org/Multi/GeneTree/Image?gt=ENSGT00390000002781. B. Survival curves of zebrafish larvae treated with control (CTRL) or p62 morpholinos and injected (or not) in the bloodstream with ≥10^3^ T3SS-ve *Shigella*. In all 4 treatments there was >90% survival. Mean±SEM of two experiments pooled, 48 larvae per group; two independent experiments. C. Bacterial counts in zebrafish larvae treated with control (CTRL) or p62 morpholinos and injected with T3SS-ve *Shigella*. Enumeration of live bacteria in homogenates from individual larvae at various times post infection measured by plating onto LB. CTRL larvae = open circles. p62-depleted larvae = open squares. Enumerations from 8 larvae per treatment, pooling of two independent experiments. Significance testing performed by Student's t test. *, P<0.05; **, P<0.01; ***, P<0.001. D. Survival curves of zebrafish larvae treated with control (CTRL) or p62 morpholinos and injected (or not) in the bloodstream with ≥10^3^ *E. coli*. In all 4 treatments there was 100% survival. n = 12 or more larvae per group; one experiment out of two. E. Bacterial counts in zebrafish larvae treated with control (CTRL) or p62 morpholinos and injected with *E. coli*. Enumeration of live bacteria in homogenates from individual larvae at various times post infection measured by plating onto LB. CTRL larvae = open circles. p62-depleted larvae = open squares. Representative enumerations from 3 larvae per treatment, one out of two independent experiments. Significance testing performed by Student's t test. **, P<0.01; ***, P<0.001.
(TIF)

Figure S6 **Infection of rapamycin-treated larvae.** A. Survival curves of zebrafish larvae treated with DMSO (CTRL) or rapamycin (RAPA) and infected (or not) in the bloodstream with ≥10^3^ T3SS- *Shigella*. In all 4 treatments there was 100% survival. n = 12 or more larvae per group; two independent experiments. B. Bacterial counts in zebrafish larvae treated with DMSO (CTRL) or rapamycin (RAPA) and infected with T3SS- *Shigella*. Enumeration of live bacteria in homogenates from individual larvae at various times post infection measured by plating onto LB. DMSO-treated larvae = open circles. RAPA-treated larvae = open squares. Enumerations from 3 larvae per treatment, two independent experiments. Mean±SEM also shown (horizontal bars). Significance testing performed by Student's t test. *, P<0.05; ***, P<0.001. C. Survival curves of zebrafish larvae treated with DMSO (CTRL) or rapamycin (RAPA) and infected (or not) in the bloodstream with ≥10^3^ *E. coli*. In all 4 treatments there was 100% survival. n = 12 or more larvae per group; one experiment out of two. D. Bacterial counts in zebrafish larvae treated with DMSO (CTRL) or rapamycin (RAPA) and infected with *E. coli*. Enumeration of live bacteria in homogenates from individual larvae at various times post infection measured by plating onto LB. DMSO-treated larvae = open circles. RAPA-treated larvae = open squares. Representative enumerations from 3 larvae per treatment, one of two independent experiments. Mean±SEM also shown (horizontal bars). Significance testing performed by Student's t test. ns, P>0.05; **, P<0.01. E. Survival curves of zebrafish larvae treated with control (CTRL) or rapamycin (RAPA) and infected with ∼1000 CFU of *L. monocytogenes* (+EGD) or not infected (no bact). n = 12 larvae per group. Significance testing performed by Log Rank test. **, P<0.01. F. Bacterial counts in zebrafish larvae treated with control (CTRL) or rapamycin (RAPA) and infected with a sublethal dose of *L. monocytogenes*. Enumeration of live bacteria in homogenates from individual larvae at various times post infection measured by plating onto BHI. CTRL larvae = open circles. RAPA-treated larvae = open squares. n = 3 larvae per treatment. Of note, dead larvae at 48 hpi (overwhelmed with bacteria) were not included here, and only larvae having survived the infection (thus far) were included here (and in this case, statistics are in grey). Mean±SEM also shown (horizontal bars). Significance testing performed by Student's t test. ns, P>0.05; *, P<0.05.
(TIF)

Video S1 **(Related to** **Fig. 3A** **). Engulfed GFP-** ***Shigella*** **persist in macrophages.** A sublethal inoculum of GFP-*Shigella* was injected in the blood and adjacent mesenchyme of a 72 hpf *mpeg1*:G/U:mCherry larva, which was live imaged every 1′07″ from 20 mpi (t = 0 on the movie) to 2h30pi (t = 2h10 on the movie) by confocal fluorescence microscopy. At the beginning of the acquisition, the injected bacteria (green) have already adhered to 6 macrophages (red, white arrows); note that neutrophils (highlighted in green by the engulfed bacteria, #1,2 and 3, yellow arrow), having engulfed similar amounts of GFP-*Shigella* as the macrophages, have already started to kill the engulfed bacteria (as indicated by the diffuse green signal) in the mesenchyme near the point of injection. At the end of the acquisition, all bacteria there have been internalized and killed by neutrophils (diffuse GFP signal, yellow arrow), while those bacteria in macrophages (4 out of 6 macrophages are still there) seem intact. The maximum intensity projection (25 planes×2 µm) is shown. Scale bar, 50 µm.
(MOV)

Video S2 **(Related to Fig. S3A). T3SS-** ***Shigella*** **are rapidly degraded by macrophages.** A 72 hpf Tg(*mpeg1*:Gal4FF^gl25^/UAS:kaede) larva was injected iv with T3SS- DsRed-*Shigella* (about 6000 to 8000 bacteria) and live imaged every 1′23″ from 20 mpi (t = 0 on the movie) until 6h20pi (t = 6h58hpi on the movie) by confocal fluorescence microscopy. 6 GFP+ macrophages harboring T3SS- DsRed-*Shigella* were tracked over time (green phagocyte with red bacteria, white arrows and labeled 1 to 6) progressively kill and degrade the engulfed bacteria (as indicated by the diffuse red label accumulating in their phagosomes) (follow single macrophage tracking on the movie). Note that T3SS- *Shigella* do not cause macrophage death nor these bacteria seem to invade the cytosol of macrophages. Note also that, at the end on the acquisition, all the T3SS- *Shigella* were cleared from the blood. The maximum intensity projection from 26 planes every 2 µm is shown. Scale bar, 20 µm.
(MOV)

Video S3 **(Related to Fig. S3A). M90T** ***Shigella*** **-dependent macrophage death.** A 72 hpf *mpeg1*:G/U:mCherry larva was injected iv with a sublethal dose of GFP-*Shigella* (about 3000 to 4000 bacteria) and live imaged every 1′55″ from 20 mpi (t = 0 on the movie) until 6h20pi (t = 6h59hpi on the movie) by confocal fluorescence microscopy. 5 mCherry+ macrophages harboring GFP-*Shigella* were tracked over time (red phagocyte with green bacteria, white arrows and labeled with number 1 to 5) progressively round up and undergoes cell death (as suggested by the loss of the red label) presumably killed by the GFP-*Shigella* (follow single macrophage tracking on the movie). Note that dying macrophages seem to burst, releasing in the extracellular milieu live GFP-*Shigella*. Note also that, at the end on the acquisition, there are still GFP-*Shigella* in the blood and adjacent mesenchyme. The maximum intensity projection from 26 planes every 2 µm is shown. Scale bar, 20 µm.
(MOV)

Video S4 **(Related to** **Fig. 3B** **). Neutrophils rapidly engulf and kill GFP-** ***Shigella*** **.** A 72 hpf *lyz*:dsRed larva was injected iv and in the adjacent mesenchyme with a sublethal inoculum of GFP-*Shigella* and live imaged by confocal fluorescence microscopy, every 2′34″ from 20 min pi (t = 0 on the movie) to 3 hpi (t = 2h37 on the movie). Neutrophils (red cell; yellow arrows follow two neutrophils over time in the movie) are recruited to the injected bacteria and efficiently phagocytose and kill them (diffuse GFP signal inside red cells), as soon as 20 mpi. Note a macrophage (white arrow, dsRed- phagocyte made visible by the engulfed green bacteria) that accumulated the GFP-*Shigella* over time (as suggested by the increase of GFP signal); by t = 1h58 it bursts and is quickly engulfed by neutrophils. At the end of the acquisition, all but few bacteria have been internalized and presumably killed by neutrophils (red cells). The maximum intensity projection (25 planes×2 µm) is shown. Scale bar, 50 µm.
(MOV)

Video S5 **(Related to** **Fig. 3C** **). GFP-** ***Shigella*** **infected macrophages eventually undergo cell death.** A 72 hpf *mpeg1*:G/U:mCherry larva was injected iv and in the mesenchyme near the injection site, with a sublethal dose of GFP-*Shigella* and live imaged every 2′12″ from 20 mpi (t = 0 on the movie) until 2h30pi (t = 2h12hpi on the movie) by confocal fluorescence microscopy. A mCherry+ macrophage harboring GFP-*Shigella* over time (red phagocyte with green bacteria, white arrow) rounds up (white arrow, t = 19h51mpi on the movie) and progressively undergoes cell death as suggested by the loss of the red label (white arrow, by t = 1h56hpi on the movie), presumably killed by the GFP-*Shigella*. Note in the bottom right corner of the entire sequence, neutrophils that have engulfed the GFP-*Shigella* kill them over time (diffuse and decreasing of the GFP intensity). The maximum intensity projection from 6 planes every 2 µm is shown. Scale bar, 10 µm.
(MOV)

Video S6 **(Related to** **Fig. 3D** **).** **Neutrophils collect dying, infected macrophages**. A sublethal dose of GFP-*Shigella* was injected in the blood and in the mesenchyme of a 72 hpf *lyz*:dsRed larva and live confocal fluorescence imaging was performed every 2′28″. The sequence shown here was started 4h20pi (t = 0 on the movie) and stopped 6 hpi (t = 1h41 on the movie). The white arrow highlights a phagocytic macrophage accumulating green bacteria (increase in the intensity of the GFP signal) over time, among dsRed+ neutrophils. By t = 1h21 this infected macrophage undergoes cell death and is rapidly engulfed by patrolling dsRed+ neutrophils (red phagocyte, yellow arrow). A single section of 2 µm is shown. Scale bar, 10 µm.
(MOV)

Video S7 **(Related to** **Fig. 3E** **). Neutrophils engulf dying, infected cells.** A sublethal dose of GFP-*Shigella* was injected in the blood and in the mesenchyme of a 72 hpf *lyz*:dsRed larva and live confocal fluorescence imaging was performed every 2′28″. The sequence shown here was acquired from 7 hpi (t = 0 on the movie) to 15 hpi (t = 9h06 on the movie). GFP-*Shigella* are able to infect other cell types (in this case a muscle fiber, area delimited by white line, decorated by green bacteria) and invade their cytoplasm, as suggested by the accumulation of GFP signal over time in this muscle fiber (see t = 1h19, t = 2h26, t = 4h08, t = 5h00 on the movie). By t = 5h49, the infected muscle fiber dies and is engulfed by dsRed+ neutrophils (red phagocytes, yellow arrows). By t = 6h34, another dying, infected muscle fiber (delimited by white line) is quickly engulfed by red neutrophils (yellow arrow). By t = 7h09, another infected, dying muscle fiber (delimitated by white line) is engulfed by red neutrophils (yellow arrow). Note that red neutrophils recruited to the site of infection efficiently engulf and clear GFP-*Shigella* over time in this sequence. A maximum intensity projection of 4 planes of 2 µm is shown. Scale bar, 10 µm.
(MOV)

Video S8 **(Related to** **Fig. 4B** **). Dynamics of** ***in vivo*** **GFP-Lc3 recruitment to DsRed-** ***Shigella*** **.** DsRed-*Shigella* escape to the cytosol and induce septin cages and autophagy in zebrafish larvae *in vivo*. GFP-Lc3 zebrafish larvae were infected subcutaneously with DsRed-*Shigella* and followed by confocal fluorescence microscopy. Single GFP-Lc3 positive leukocytes accumulating GFP-lc3 around *Shigella* are tracked by arrows; single GFP-Lc3 positive leukocytes having engulfed the bacteria without GFP-Lc3 accumulation are highlighted by white circles. The movie starts 3h10min pi (T = 0 on the movie) and shows 2h50min acquisition. Time lapse every 1 min. The maximum intensity projection from 7 planes every 2 µm is shown. Scale bar, 20 µm.
(MOV)

Video S9 **(Related to** **Fig. 4C** **). Tilt series of** ***Shigella*** **autophagy** ***in vivo*** **.** Cytosolic *Shigella* are sequestered in autophagosomes *in vivo*. Zebrafish larvae were infected in the tail muscle with GFP-*Shigella* for 4 h (medium dose) and fixed for EM. Scale bar, 0.5 µm.
(MOV)

Video S10 **(Related to Fig. S4B). Dynamics of** ***in vivo*** **GFP-Lc3 recruitment to DsRed-** ***M. marinum.*** *M. marinum* escape to the cytosol and induce septin cages and autophagy in zebrafish larvae *in vivo*. GFP-Lc3 zebrafish larvae were infected subcutaneously with DsRed-*M. marinum* and followed by confocal fluorescence microscopy. Shown here is an example of an GFP-Lc3 positive leukocyte (arrow) controlling *M. marinum* and progressively accumulating GFP-Lc3 to the internalized bacteria. In the same field, note two GFP-Lc3 positive leukocytes (white circles) that do not accumulate GFP-Lc3 to the internalized bacteria, and that presumably do not control intracellular *M. marinum* proliferation. The movie starts 1h10mpi (T = 0 on the movie) and ends 2h30mpi. Time lapse every 2′30″. The maximum intensity projection from 5 planes every 2 µm is shown. Scale bar, 20 µm.
(MOV)

## References

[1] Mizushima N, Yoshimori T, Ohsumi Y (2011) The role of Atg proteins in autophagosome formation. Annu Rev Cell Dev Biol 27: 107–132. 21801009 10.1146/annurev-cellbio-092910-154005

[2] Levine B, Mizushima N, Virgin HW (2011) Autophagy in immunity and inflammation. Nature 469: 323–335. 21248839 10.1038/nature09782 PMC3131688

[3] Mizushima N, Komatsu M (2011) Autophagy: renovation of cells and tissues. Cell 147: 728–741. 22078875 10.1016/j.cell.2011.10.026

[4] Johansen T, Lamark T (2011) Selective autophagy mediated by autophagic adapter proteins. Autophagy 7: 279–296. 21189453 10.4161/auto.7.3.14487 PMC3060413

[5] Kraft C, Peter M, Hofmann K (2010) Selective autophagy: ubiquitin-mediated recognition and beyond. Nat Cell Biol 12: 836–841. 20811356 10.1038/ncb0910-836

[6] Pankiv S, Clausen TH, Lamark T, Brech A, Bruun J-A, et al (2007) p62/SQSTM1 binds directly to Atg8/LC3 to facilitate degradation of ubiquitinated protein aggregates by autophagy. J Biol Chem 282: 24131–24145. 17580304 10.1074/jbc.M702824200

[7] Deretic V (2012) Autophagy as an innate immunity paradigm: expanding the scope and repertoire of pattern recognition receptors. Curr Opin Immunol 24: 21–31. 22118953 10.1016/j.coi.2011.10.006 PMC3288884

[8] Ogawa M, Yoshimori T, Suzuki T, Sagara H, Mizushima N, et al (2005) Escape of intracellular *Shigella* from autophagy. Science 307: 727–731. 15576571 10.1126/science.1106036

[9] Py BF, Lipinski MM, Yuan J (2007) Autophagy limits *Listeria monocytogenes* intracellular growth in the early phase of primary infection. Autophagy 3: 117–125. 17204850 10.4161/auto.3618

[10] Birmingham CL, Smith AC, Bakowski MA, Yoshimori T, Brumell JH (2006) Autophagy controls *Salmonella* infection in response to damage to the *Salmonella*-containing vacuole. J Biol Chem 281: 11374–11383. 16495224 10.1074/jbc.M509157200

[11] Gutierrez MG, Master SS, Singh SB, Taylor GA, Colombo MI, et al (2004) Autophagy is a defense mechanism inhibiting BCG and *Mycobacterium tuberculosis* survival in infected macrophages. Cell 119: 753–766. 15607973 10.1016/j.cell.2004.11.038

[12] Mostowy S, Cossart P (2012) Bacterial autophagy: restriction or promotion of bacterial replication? Trend Cell Biol 22: 283–291. 10.1016/j.tcb.2012.03.006 22555009

[13] Mostowy S (2013) Autophagy and bacterial clearance: a not so clear picture. Cellular Microbiol 15: 395–402. 10.1111/cmi.12063 PMC3592990 23121192

[14] Mostowy S, Sancho-Shimizu V, Hamon M, Simeone R, Brosch R, et al (2011) p62 and NDP52 proteins target intracytosolic *Shigella* and *Listeria* to different autophagy pathways. J Biol Chem 286: 26987–26995. 21646350 10.1074/jbc.M111.223610 PMC3143657

[15] Mostowy S, Bonazzi M, Hamon MA, Tham TN, Mallet A, et al (2010) Entrapment of intracytosolic bacteria by septin cage-like structures. Cell Host Microbe 8: 433–444. 21075354 10.1016/j.chom.2010.10.009

[16] Ogawa M, Yoshikawa Y, Kobayashi T, Mimuro H, Fukumatsu M, et al (2011) A Tecpr1-dependent selective autophagy pathway targets bacterial pathogens. Cell Host Microbe 9: 376–389. 21575909 10.1016/j.chom.2011.04.010

[17] Mostowy S, Cossart P (2012) Septins: the fourth component of the cytoskeleton. Nat Rev Mol Cell Biol 13: 183–194. 22314400 10.1038/nrm3284

[18] Shim D-H, Suzuki T, Chang S-Y, Park S-M, Sansonetti PJ, et al (2007) New animal model of shigellosis in the guinea pig: its usefulness for protective efficacy studies. J Immunol 178: 2476–2482. 17277155 10.4049/jimmunol.178.4.2476

[19] Perdomo OJ, Cavaillon JM, Huerre M, Ohayon H, Gounon P, et al (1994) Acute inflammation causes epithelial invasion and mucosal destruction in experimental shigellosis. J Exp Med 180: 1307–1319. 7931064 10.1084/jem.180.4.1307 PMC2191671

[20] Lieschke GJ, Trede NS (2009) Fish immunology. Curr Biol 19: R678–R682. 19706273 10.1016/j.cub.2009.06.068

[21] Renshaw SA, Trede NS (2012) A model 450 million years in the making: zebrafish and vertebrate immunity. Dis Model Mech 5: 38–47. 22228790 10.1242/dmm.007138 PMC3255542

[22] Santoriello C, Zon LI (2012) Hooked! Modeling human disease in zebrafish. J Clin Invest 122: 2337–2343. 22751109 10.1172/JCI60434 PMC3386812

[23] Herbomel P, Thisse B, Thisse C (1999) Ontogeny and behaviour of early macrophages in the zebrafish embryo. Development 126: 3735–3745. 10433904 10.1242/dev.126.17.3735

[24] Levraud JP, Disson O, Kissa K, Bonne I, Cossart P, et al (2009) Real-time observation of *Listeria monocytogenes*-phagocyte interactions in living zebrafish larvae. Infect Immun 77: 3651–3660. 19546195 10.1128/IAI.00408-09 PMC2738018

[25] Davis JM, Clay H, Lewis JL, Ghori N, Herbomel P, et al (2002) Real-time visualization of mycobacterium-macrophage interactions leading to initiation of granuloma formation in zebrafish embryos. Immunity 17: 693–702. 12479816 10.1016/s1074-7613(02)00475-2

[26] Colucci-Guyon E, Tinevez J-Y, Renshaw SA, Herbomel P (2011) Strategies of professional phagocytes in vivo: unlike macrophages, neutrophils engulf only surface-associated microbes. J Cell Sci 124: 3053–3059. 21868367 10.1242/jcs.082792

[27] Van Der Sar AM, Musters RJP, Van Eeden FJM, Appelmelk BJ, Vandenbroucke-Grauls CMJE, et al (2003) Zebrafish embryos as a model host for the real time analysis of *Salmonella* Typhimurium infections. Cell Microbiol 5: 601–611. 12925130 10.1046/j.1462-5822.2003.00303.x

[28] Clatworthy AE, Lee JS-W, Leibman M, Kostun Z, Davidson AJ, et al (2009) *Pseudomonas aeruginosa* infection of zebrafish involves both host and pathogen determinants. Infect Immun 77: 1293–1303. 19168742 10.1128/IAI.01181-08 PMC2663173

[29] Brannon MK, Davis JM, Mathias JR, Hall CJ, Emerson JC, et al (2009) *Pseudomonas aeruginosa* Type III secretion system interacts with phagocytes to modulate systemic infection of zebrafish embryos. Cell Microbiol 11: 755–768. 19207728 10.1111/j.1462-5822.2009.01288.x PMC2933946

[30] Vergunst AC, Meijer AH, Renshaw SA, O'Callaghan D (2010) *Burkholderia cenocepacia* creates an intramacrophage replication niche in zebrafish embryos, followed by bacterial dissemination and establishment of systemic infection. Infect Immun 78: 1495–1508. 20086083 10.1128/IAI.00743-09 PMC2849400

[31] Prajsnar TK, Hamilton R, Garcia-Lara J, McVicker G, Williams A, et al (2012) A privileged intraphagocyte niche is responsible for disseminated infection of *Staphylococcus aureus* in a zebrafish model. Cell Microbiol 14: 1600–1619. 22694745 10.1111/j.1462-5822.2012.01826.x PMC3470706

[32] Harvie EA, Green JM, Neely MN, Huttenlocher A (2013) Innate immune response to *Streptococcus iniae* infection in zebrafish larvae. Infect Immun 81: 110–121. 23090960 10.1128/IAI.00642-12 PMC3536132

[33] Kanther M, Rawls JF (2010) Host–microbe interactions in the developing zebrafish. Curr Opin Immunol 22: 10–19. 20153622 10.1016/j.coi.2010.01.006 PMC3030977

[34] Perrin AJ, Jiang X, Birmingham CL, So NSY, Brumell JH (2004) Recognition of bacteria in the cytosol of mammalian cells by the ubiquitin system. Curr Biol 14: 806–811. 15120074 10.1016/j.cub.2004.04.033

[35] Collins CA, De Mazière A, van Dijk S, Carlsson F, Klumperman J, et al (2009) Atg5-independent sequestration of ubiquitinated mycobacteria. PLoS Pathog 5: e1000430. 19436699 10.1371/journal.ppat.1000430 PMC2673685

[36] Yoshikawa Y, Ogawa M, Hain T, Yoshida M, Fukumatsu M, et al (2009) *Listeria monocytogenes* ActA-mediated escape from autophagic recognition. Nat Cell Biol 11: 1233–1240. 19749745 10.1038/ncb1967

[37] Pan F, Malmberg R, Momany M (2007) Analysis of septins across kingdoms reveals orthology and new motifs. BMC Evol Biol 7: 103. 17601340 10.1186/1471-2148-7-103 PMC1931588

[38] Mostowy S, Nam Tham T, Danckaert A, Guadagnini S, Boisson-Dupuis S, et al (2009) Septins regulate bacterial entry into host cells. PLoS ONE 4: e4196. 19145258 10.1371/journal.pone.0004196 PMC2626286

[39] Kinoshita M, Field CM, Coughlin ML, Straight AF, Mitchison TJ (2002) Self- and actin-templated assembly of mammalian septins. Dev Cell 3: 791–802. 12479805 10.1016/s1534-5807(02)00366-0

[40] Le Guyader D, Redd MJ, Colucci-Guyon E, Murayama E, Kissa K, et al (2008) Origins and unconventional behavior of neutrophils in developing zebrafish. Blood 111: 132–141. 17875807 10.1182/blood-2007-06-095398

[41] Zychlinsky A, Prevost MC, Sansonetti PJ (1992) *Shigella flexneri* induces apoptosis in infected macrophages. Nature 358: 167–169. 1614548 10.1038/358167a0

[42] Cossart P, Sansonetti PJ (2004) Bacterial invasion: the paradigms of enteroinvasive pathogens. Science 304: 242–248. 15073367 10.1126/science.1090124

[43] Klionsky D (2012) Guidelines for the use and interpretation of assays for monitoring autophagy in higher eukaryotes. Autophagy 4: 151–75. 10.4161/auto.5338 PMC2654259 18188003

[44] Mizushima N, Yoshimori T, Levine B (2010) Methods in mammalian autophagy research. Cell 140: 313–326. 20144757 10.1016/j.cell.2010.01.028 PMC2852113

[45] Laplante M, Sabatini David M (2012) mTOR signaling in growth control and disease. Cell 149: 274–293. 22500797 10.1016/j.cell.2012.03.017 PMC3331679

[46] He C, Klionsky DJ (2010) Analyzing autophagy in zebrafish. Autophagy 6: 642–644. 20495344 10.4161/auto.6.5.12092 PMC3654832

[47] Boglev Y, Badrock AP, Trotter AJ, Du Q, Richardson EJ, et al (2013) Autophagy induction is a Tor- and Tp53-independent cell survival response in a zebrafish model of disrupted ribosome biogenesis. PLoS Genet 9: e1003279. 23408911 10.1371/journal.pgen.1003279 PMC3567153

[48] Makky K, Tekiela J, Mayer AN (2007) Target of rapamycin (TOR) signaling controls epithelial morphogenesis in the vertebrate intestine. Dev Biol 303: 501–513. 17222402 10.1016/j.ydbio.2006.11.030 PMC2715143

[49] Deretic V, Levine B (2009) Autophagy, immunity, and microbial adaptations. Cell Host Microbe 5: 527–549. 19527881 10.1016/j.chom.2009.05.016 PMC2720763

[50] Hall Christopher J, Flores Maria V, Oehlers Stefan H, Sanderson Leslie E, Lam Enid Y, et al (2012) Infection-responsive expansion of the hematopoietic stem and progenitor cell compartment in zebrafish Is dependent upon inducible nitric oxide. Cell Stem Cell 10: 198–209. 22305569 10.1016/j.stem.2012.01.007

[51] Zhu L, Zhao G, Stein R, Zheng X, Hu W, et al (2010) The proteome of *Shigella flexneri* 2a 2457T grown at 30 and 37°C. Mol Cell Proteomics 9: 1209–1220. 20164057 10.1074/mcp.M900446-MCP200 PMC2877981

[52] Prajsnar TK, Cunliffe VT, Foster SJ, Renshaw SA (2008) A novel vertebrate model of *Staphylococcus aureu*s infection reveals phagocyte-dependent resistance of zebrafish to non-host specialized pathogens. Cell Microbiol 10: 2312–2325. 18715285 10.1111/j.1462-5822.2008.01213.x

[53] Holub M, Cheng C-W, Mott S, Wintermeyer P, van Rooijen N, et al (2009) Neutrophils sequestered in the liver suppress the proinflammatory response of Kupffer cells to systemic bacterial infection. J Immunol 183: 3309–3316. 19641138 10.4049/jimmunol.0803041

[54] Suzuki T, Franchi L, Toma C, Ashida H, Ogawa M, et al (2007) Differential regulation of caspase-1 activation, pyroptosis, and autophagy via Ipaf and ASC in *Shigella*-infected macrophages. PLoS Pathog 3: e111. 17696608 10.1371/journal.ppat.0030111 PMC1941748

[55] Yang CT, Cambier CJ, Davis JM, Hall Christopher J, Crosier Philip S, et al (2012) Neutrophils exert protection in the early tuberculous granuloma by oxidative killing of mycobacteria phagocytosed from infected macrophages. Cell Host Microbe 12: 301–312. 22980327 10.1016/j.chom.2012.07.009 PMC3638950

[56] Tattoli I, Sorbara MT, Vuckovic D, Ling A, Soares F, et al (2012) Amino acid starvation induced by invasive bacterial pathogens triggers an innate host defense program. Cell Host Microbe 11: 563–575. 22704617 10.1016/j.chom.2012.04.012

[57] Ellett F, Pase L, Hayman JW, Andrianopoulos A, Lieschke GJ (2011) mpeg1 promoter transgenes direct macrophage-lineage expression in zebrafish. Blood 117: e49–e56. 21084707 10.1182/blood-2010-10-314120 PMC3056479

[58] Gray C, Loynes CA, Whyte MK, Crossman DC, Renshaw SA, et al (2011) Simultaneous intravital imaging of macrophage and neutrophil behaviour during inflammation using a novel transgenic zebrafish. Thromb Haemostasis 105: 811–819. 21225092 10.1160/TH10-08-0525

[59] Hall C, Flores M, Storm T, Crosier K, Crosier P (2007) The zebrafish lysozyme C promoter drives myeloid-specific expression in transgenic fish. BMC Dev Biol 7: 42. 17477879 10.1186/1471-213X-7-42 PMC1877083

[60] Westerfield M (1993) The zebrafish book. A guide for the laboratory use of zebrafish (*Brachydanio rerio*). Eugene, Oregon: University of Oregon Press, Eugene.

[61] Kimmel C, Ballard W, Kimmel S, Ullmann B, Schilling T (1995) Stages of embryonic development of the zebrafish. Dev Dyn 203: 253–310. 8589427 10.1002/aja.1002030302

[62] Salgado-Pabón W, Celli S, Arena ET, Nothelfer K, Roux P, et al (2013) Shigella impairs T lymphocyte dynamics in vivo. Proc Natl Acad Sci USA 110: 4458–4463. 23417297 10.1073/pnas.1300981110 PMC3606969

[63] Gibbings D, Mostowy S, Jay F, Schwab Y, Cossart P, et al (2012) Selective autophagy degrades DICER and AGO2 and regulates miRNA activity. Nat Cell Biol 14: 1314–1321. 23143396 10.1038/ncb2611 PMC3771578

[64] Hollinshead M, Johns HL, Sayers CL, Gonzalez-Lopez C, Smith GL, et al (2012) Endocytic tubules regulated by Rab GTPases 5 and 11 are used for envelopment of herpes simplex virus. EMBO J 31: 4204–4220. 22990238 10.1038/emboj.2012.262 PMC3492727

[65] Lutfalla G, Uze G (2006) Performing quantitative reverse-transcribed polymerase chain reaction experiments. In: Alan K, Brian O, editors. Methods in Enzymology: Academic Press. pp. 386–400. 10.1016/S0076-6879(06)10019-1 16938562

[66] Ludwig M, Palha N, Torhy C, Briolat V, Colucci-Guyon E, et al (2011) Whole-body analysis of a viral infection: vascular endothelium is a primary target of infectious hematopoietic necrosis virus in zebrafish larvae. PLoS Pathog 7: e1001269. 21304884 10.1371/journal.ppat.1001269 PMC3033377

