# Supplementary figures and images for: The Zebrafish as a New Model for the In Vivo Study of Shigella flexneri Interaction with Phagocytes and Bacterial Autophagy
Source: PLoS Pathog. 2013 Sep 5;9(9):e1003588. doi: 10.1371/journal.ppat.1003588 (PMC3764221; doi:10.1371/journal.ppat.1003588)

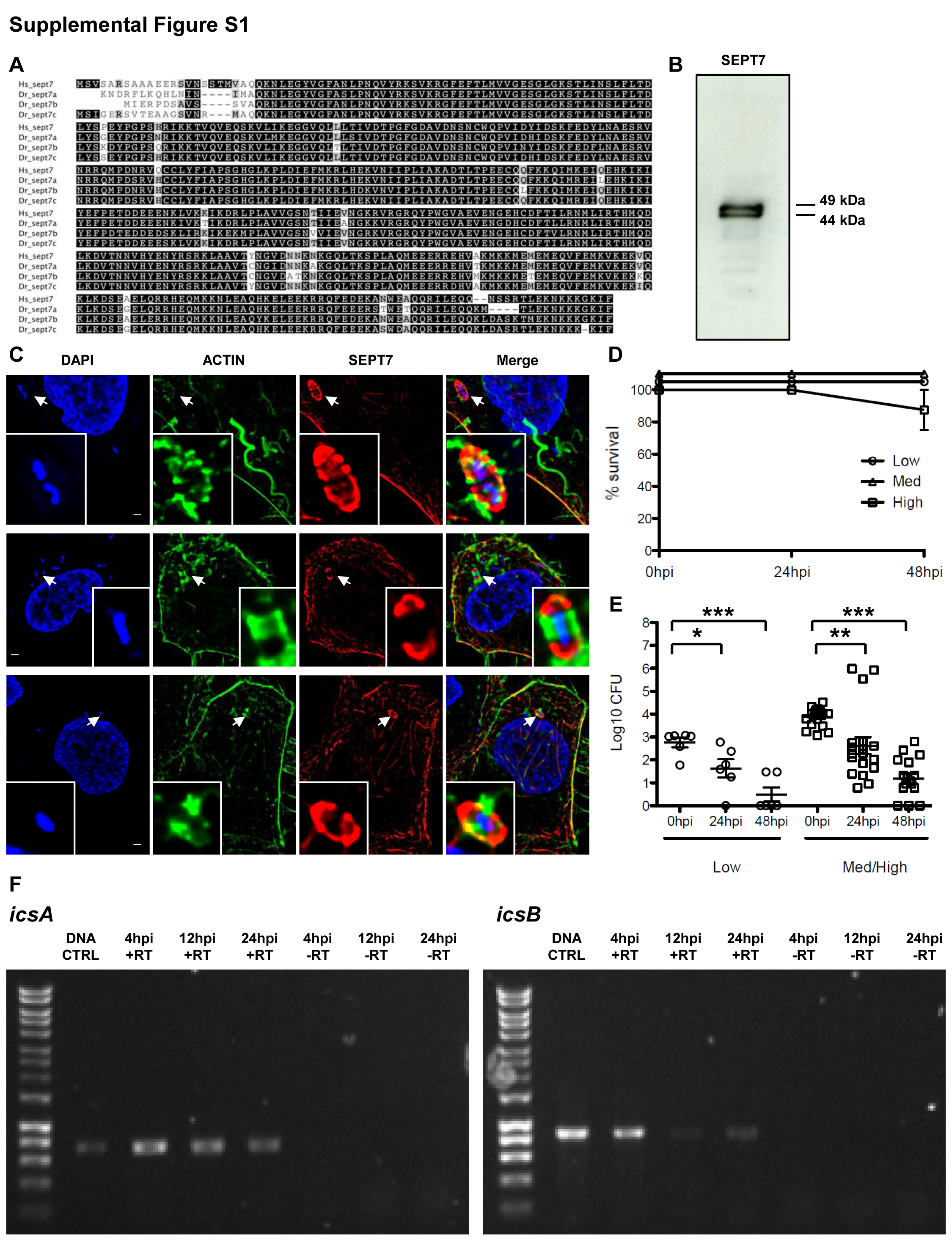

Supplement: Figure S1 — Characterization of zebrafish septin 7 and Shigella virulence factors. A. Alignment of zebrafish and human septin 7 proteins. Human septin 7 (NM_001788.5) and its three zebrafish orthologues: sept7a (deduced from a sequence we re-amplified and cloned because of inconsistencies in the available sequences and deposited at GenBank KC513820, gene ENSDARG00000052673 on chromosome 16), sept7b (NP_001071211, gene ENSDARG00000019649 on chromosome 25) and sept7c (NP_001242958, gene located on chromosome 19 in a region improperly assembled on zv9, broken in three parts: ENSDART00000126747, ENSDARG00000075198 and ENSDART00000131014). B. Western blot of larval extracts using antibodies against SEPT7 as described in Fig. 1A. Shown here is complete lane of SEPT7 blot and antibody specificity. C. Shigella forms actin tails and recruits septin cages in HeLa cells at 28°C. Immunofluoresence microscopy of HeLa cells infected with Shigella for 4h40min at 28°C. F-actin (green), SEPT7 (red), DAPI (blue). Note septin cage-like structures surrounding DAPI-labeled Shigella (arrows). Scale bar, 2 µm. D. Survival curves of 72 hpf larvae injected with various doses of T3SS- S. flexneri and incubated at 28°C for 48 hpi. The effective inoculum, quantified a posteriori, was classified as low (<103 CFU, open circles; effective range: 0.6–11.7×102 CFU), medium (∼4×10^3^ CFU, open triangles; effective range: 1.2–6.0×10^3^ CFU) or high (∼104 CFU, open squares; effective range: 6.1–33.3×103 CFU). Mean±SEM of 24 larvae from 2 independent experiments per inoculum class. E. Enumeration of live bacteria (T3SS- Shigella) in homogenates from individual larvae at various times post infection measured by plating onto LB. Low dose inoculum = open circles. Medium or high dose inoculum = open squares. Mean±SEM also shown (horizontal bars). Significance testing performed by Student's t test. *, P<0.05; **, P<0.01; ***, P<0.001. F. icsA and icsB expression in vivo. S. flexneri M90T was injected into the hindbrain ven [file ppat.1003588.s001.tif]

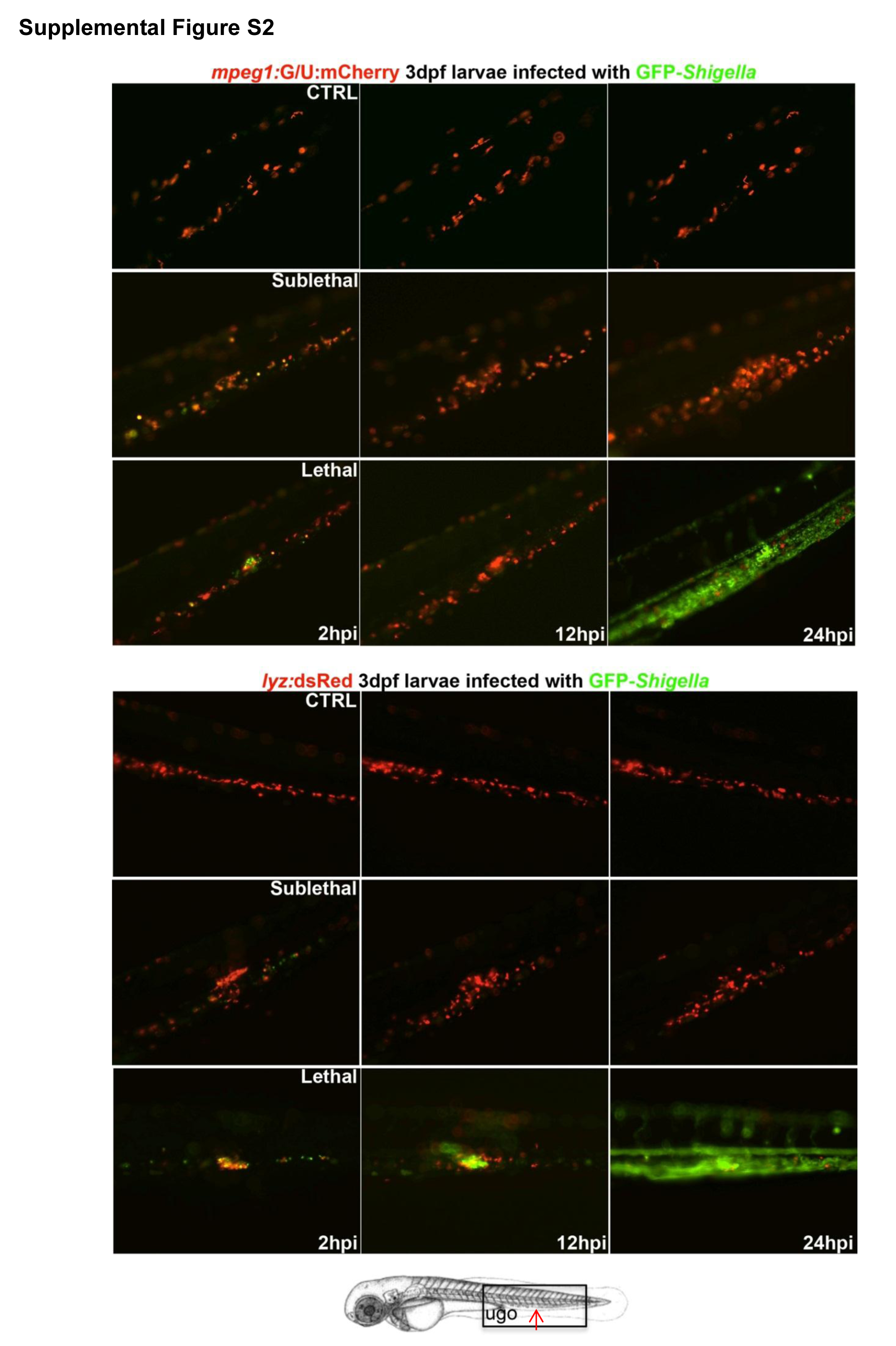

Supplement: Figure S2 — In vivo macrophage and neutrophil behaviours and progression of the infection over time upon sublethal and lethal Shigella inocula. Images of the caudal area of Mpeg1:G/U:mCherry (red macrophages, upper panel) or lyz:dsRed (red neutrophils, lower panel) 3 dpf larvae iv injected with sublethal and lethal GFP-Shigella inocula. Live imaging by widefield microscopy, overlay of red and green fluorescence. Images at 2, 12 and 24 hpi are shown. (Bottom) Scheme of a 72 hpf larva: the imaged region is boxed, and arrow shows the injection site. (TIF) [file ppat.1003588.s002.tif]

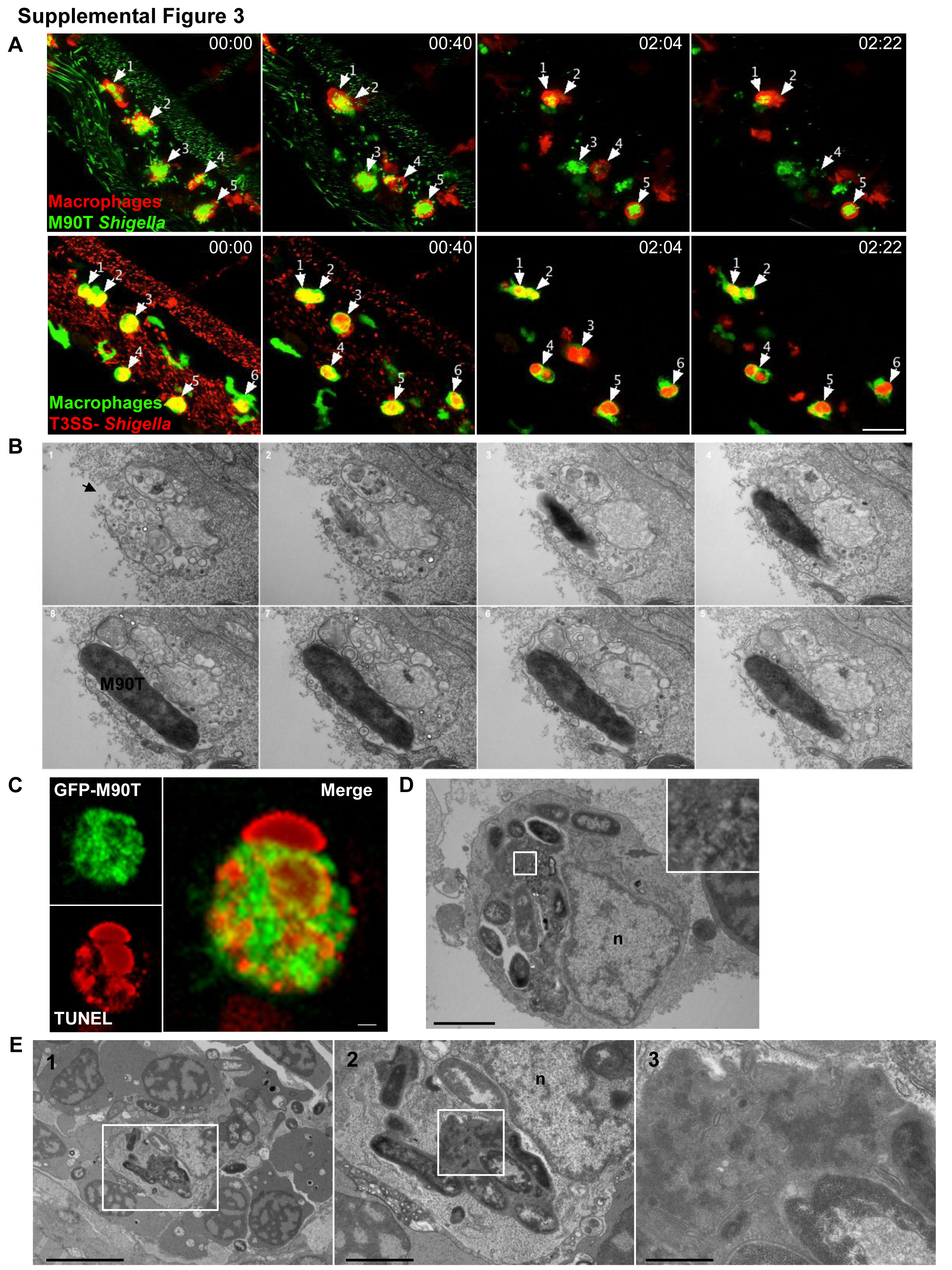

Supplement: Figure S3 — Characterisation of Shigella -dependent macrophage death. A. Frames extracted from in vivo time-lapse confocal imaging sessions of 3 dpf larvae injected in the bloodstream with GFP-Shigella (3000–4000 bacteria) and T3SS- DsRed Shigella (6000–8000 bacteria). Caudal area, rostral to bottom right, dorsal to top right. Maximum intensity projection from 26 planes every 2 µm. Scale bar, 20 µm. Upper panel: mpeg1:G/U:mCherry larva (red macrophages); first frame at 20 mpi. By 20 mpi, GFP-Shigella have already adhered to or have been engulfed by macrophages (red cells, white arrows, number 1 to 5). Tracked infected macrophages are killed by GFP-Shigella; they progressively round up and burst, releasing in the extracellular milieu live GFP-Shigella. Note that at the end of the sequence (about 3 hpi) the bacteria are still in the blood. See also Video S3. Lower panel: 72 hpf Tg(mpeg1:Gal4FFgl25/UAS:kaede) larva (green macrophages); first frame at 20 mpi. By 20 mpi T3SS- DsRed-Shigella have been engulfed by macrophages (white arrow, number 1 to 6). Tracked infected macrophages progressively kill the engulfed bacteria (diffuse red staining accumulating in their phagosomes) over time. Note that at the end of the sequence (about 3 hpi) all the red bacteria have been cleared from the blood, without any sign of cell death. See also Video S2. B. Bacteria are killing host cells (leukocytes) in the caudal vein. From the ultrastructure of the infected dying cell, plasma membrane rupture and damaged cellular material are observed, hallmarks of pyroptosis. Zebrafish larvae were infected in the tail muscle with GFP-Shigella (medium dose) for 4 h and fixed for EM. Shown here are 8 serial sections in a part of infected larvae. The first section shows the plasma membrane is ruptured (black arrow) and the cellular material is damaged. In the remaining sections, a bacterium (labeled in frame 5 as M90T) is inside the damaged cell. Scale bar, 0.5 µm. C. Infected cells are dying as shown by TUNEL [file ppat.1003588.s003.tif]

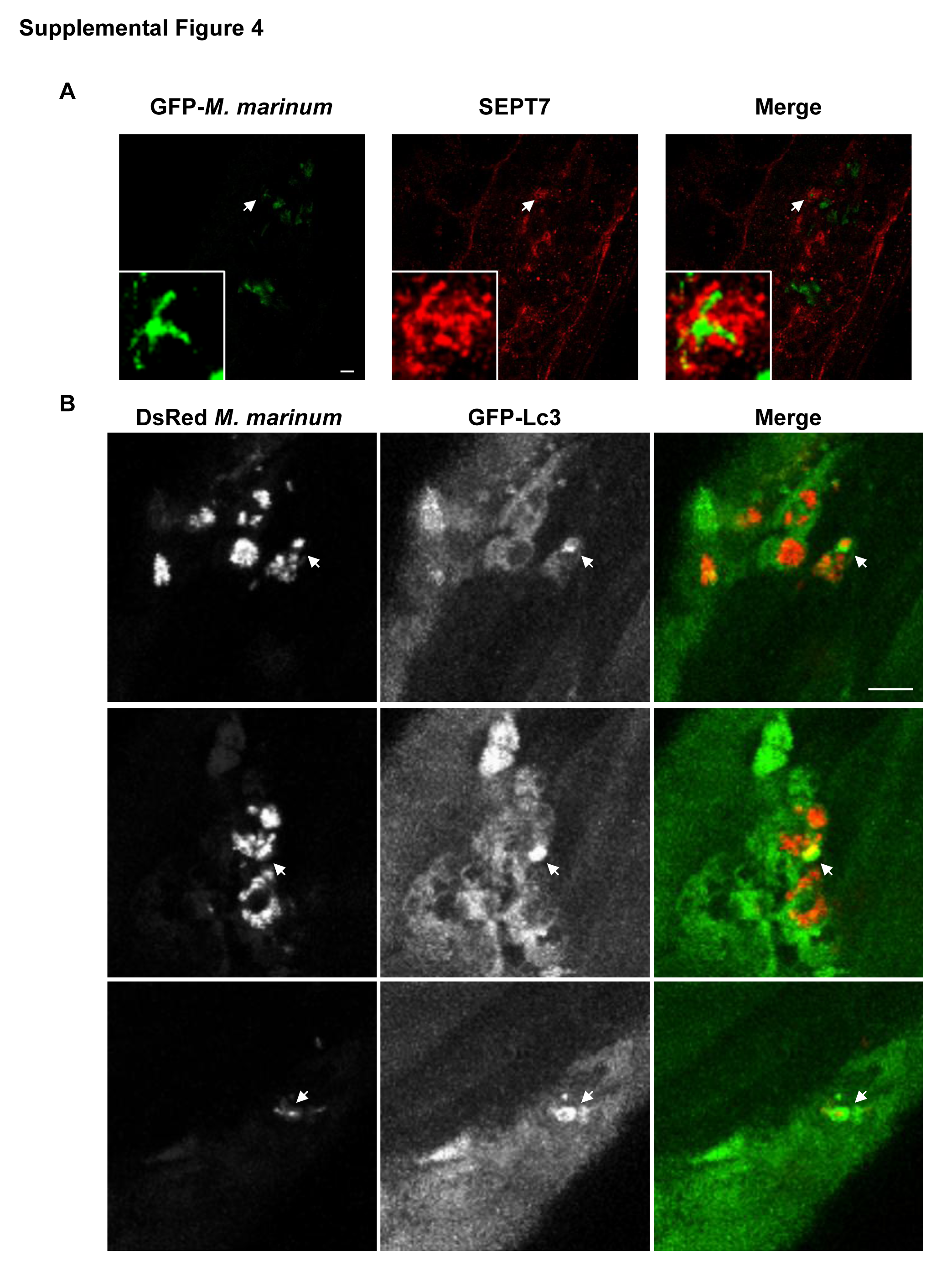

Supplement: Figure S4 — M. marinum escape to the cytosol and induce septin caging and autophagy in zebrafish larvae in vivo . A. Zebrafish larvae were infected subcutaneously with GFP-M. marinum for 48 h (low dose), fixed, labeled with antibodies against SEPT7 (red) and to GFP (green) and imaged by confocal microscopy. Scale bar, 5 µm. B. GFP-Lc3 zebrafish larvae were infected subcutaneously with DsRed-M. marinum and live imaged by confocal microscopy. Shown here is an example of 3 GFP-Lc3 positive leukocytes having engulfed M. marinum and highlights Lc3 recruitment to bacteria. The maximum intensity projection from 5 planes every 2 µm is shown. Scale bar, 10 µm. See also Video S10. (TIF) [file ppat.1003588.s004.tif]

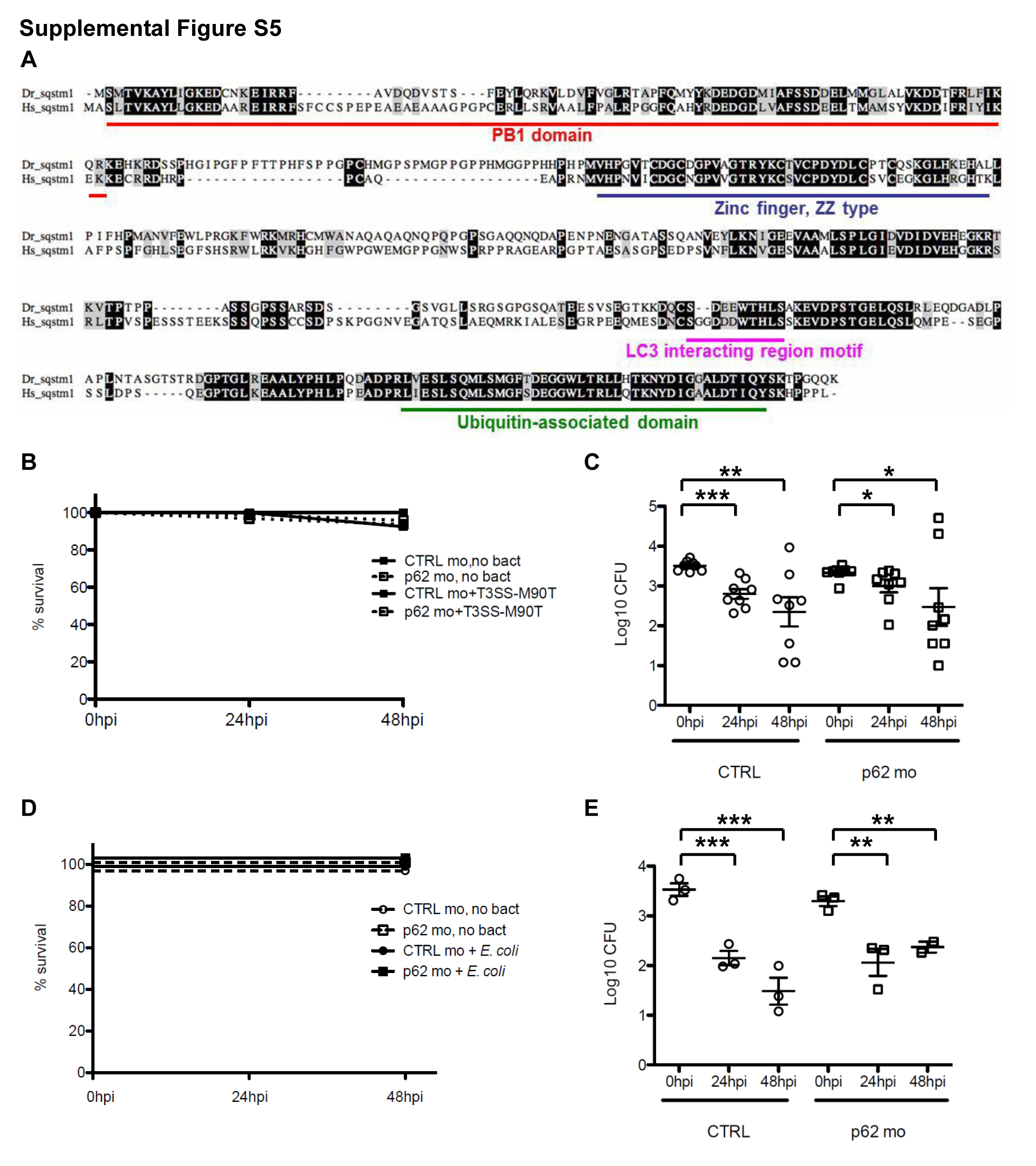

Supplement: Figure S5 — Infection of p62-depleted larvae. A. Alignment of zebrafish and human p62. Alignment of human p62/sqstm1 (NP_003891) and its zebrafish orthologue (gene ENSDARG00000075014 on chromosome 14; cDNA sequence deduced from EST consensus and deposited at GenBank KC513821). Analysis by Ensembl Genetree shows p62/sqstm1 is highly conserved between human and zebrafish: http://www.ensembl.org/Multi/GeneTree/Image?gt=ENSGT00390000002781. B. Survival curves of zebrafish larvae treated with control (CTRL) or p62 morpholinos and injected (or not) in the bloodstream with ≥103 T3SS-ve Shigella. In all 4 treatments there was >90% survival. Mean±SEM of two experiments pooled, 48 larvae per group; two independent experiments. C. Bacterial counts in zebrafish larvae treated with control (CTRL) or p62 morpholinos and injected with T3SS-ve Shigella. Enumeration of live bacteria in homogenates from individual larvae at various times post infection measured by plating onto LB. CTRL larvae = open circles. p62-depleted larvae = open squares. Enumerations from 8 larvae per treatment, pooling of two independent experiments. Significance testing performed by Student's t test. *, P<0.05; **, P<0.01; ***, P<0.001. D. Survival curves of zebrafish larvae treated with control (CTRL) or p62 morpholinos and injected (or not) in the bloodstream with ≥103 E. coli. In all 4 treatments there was 100% survival. n = 12 or more larvae per group; one experiment out of two. E. Bacterial counts in zebrafish larvae treated with control (CTRL) or p62 morpholinos and injected with E. coli. Enumeration of live bacteria in homogenates from individual larvae at various times post infection measured by plating onto LB. CTRL larvae = open circles. p62-depleted larvae = open squares. Representative enumerations from 3 larvae per treatment, one out of two independent experiments. Significance testing performed by Student's t test. **, P<0.01; ***, P<0.001. (TIF) [file ppat.1003588.s005.tif]

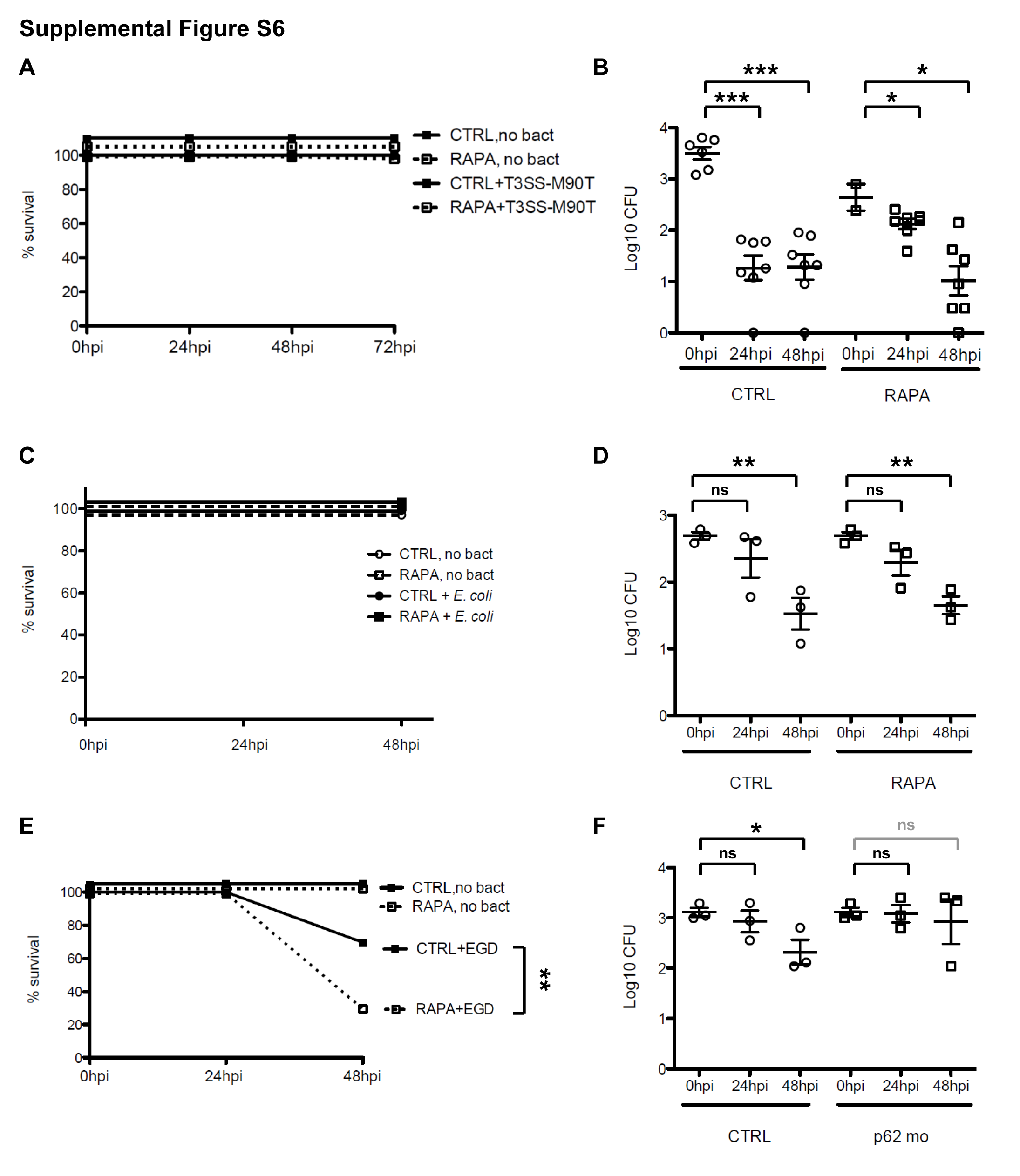

Supplement: Figure S6 — Infection of rapamycin-treated larvae. A. Survival curves of zebrafish larvae treated with DMSO (CTRL) or rapamycin (RAPA) and infected (or not) in the bloodstream with ≥103 T3SS- Shigella. In all 4 treatments there was 100% survival. n = 12 or more larvae per group; two independent experiments. B. Bacterial counts in zebrafish larvae treated with DMSO (CTRL) or rapamycin (RAPA) and infected with T3SS- Shigella. Enumeration of live bacteria in homogenates from individual larvae at various times post infection measured by plating onto LB. DMSO-treated larvae = open circles. RAPA-treated larvae = open squares. Enumerations from 3 larvae per treatment, two independent experiments. Mean±SEM also shown (horizontal bars). Significance testing performed by Student's t test. *, P<0.05; ***, P<0.001. C. Survival curves of zebrafish larvae treated with DMSO (CTRL) or rapamycin (RAPA) and infected (or not) in the bloodstream with ≥103 E. coli. In all 4 treatments there was 100% survival. n = 12 or more larvae per group; one experiment out of two. D. Bacterial counts in zebrafish larvae treated with DMSO (CTRL) or rapamycin (RAPA) and infected with E. coli. Enumeration of live bacteria in homogenates from individual larvae at various times post infection measured by plating onto LB. DMSO-treated larvae = open circles. RAPA-treated larvae = open squares. Representative enumerations from 3 larvae per treatment, one of two independent experiments. Mean±SEM also shown (horizontal bars). Significance testing performed by Student's t test. ns, P>0.05; **, P<0.01. E. Survival curves of zebrafish larvae treated with control (CTRL) or rapamycin (RAPA) and infected with ∼1000 CFU of L. monocytogenes (+EGD) or not infected (no bact). n = 12 larvae per group. Significance testing performed by Log Rank test. **, P<0.01. F. Bacterial counts in zebrafish larvae treated with control (CTRL) or rapamycin (RAPA) and infected with a sublethal dose of L. monocytogenes. Enumeration of live bacteria [file ppat.1003588.s006.tif]
